# Recent Advances in Strain-Induced Piezoelectric and Piezoresistive Effect-Engineered 2D Semiconductors for Adaptive Electronics and Optoelectronics

**DOI:** 10.1007/s40820-020-00439-9

**Published:** 2020-05-04

**Authors:** Feng Li, Tao Shen, Cong Wang, Yupeng Zhang, Junjie Qi, Han Zhang

**Affiliations:** 1 Institute of Microscale Optoelectronics, International Collaborative Laboratory of 2D Materials for Optoelectronics Science and Technology of Ministry of Education, College of Physics and Optoelectronic Engineering, Shenzhen University, Shenzhen, 518060 People’s Republic of China; 2 School of Materials Science and Engineering, University of Science and Technology Beijing, Beijing, 100083 People’s Republic of China

**Keywords:** 2D semiconductors, Strain, Piezoelectric effect, Piezoresistive effect, Electronic and optoelectronics

## Abstract

The development of two-dimensional (2D) semiconductors has attracted widespread attentions in the scientific community and industry due to their ultra-thin thickness, unique structure, excellent optoelectronic properties and novel physics. The excellent flexibility and outstanding mechanical strength of 2D semiconductors provide opportunities for fabricated strain-sensitive devices and utilized strain tuning their electronic and optic–electric performance. The strain-engineered one-dimensional materials have been well investigated, while there is a long way to go for 2D semiconductors. In this review, starting with the fundamental theories of piezoelectric and piezoresistive effect resulted by strain, following we reviewed the recent simulation works of strain engineering in novel 2D semiconductors, such as Janus 2D and 2D-Xene structures. Moreover, recent advances in experimental observation of strain tuning PL spectra and transport behavior of 2D semiconductors are summarized. Furthermore, the applications of strain-engineered 2D semiconductors in sensors, photodetectors and nanogenerators are also highlighted. At last, we in-depth discussed future research directions of strain-engineered 2D semiconductor and related electronics and optoelectronics device applications.

## Highlights

- A comprehensive review of strain-engineered 2D semiconductors in electronics and optoelectronics. The basic theories and simulation studies of strain introduced piezoelectric effect and piezoresistive effect have been summarized.
- The various experimental methods for study strain-engineered 2D semiconductors have been highlighted.
- The applications of strain sensor, strain tuning the performance of photodetector and piezoelectric nanogenerator have been reviewed.

## Graphical abstract

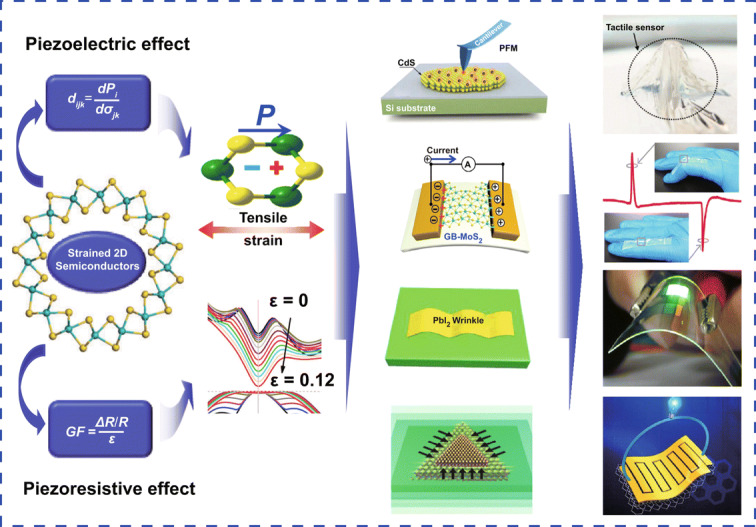

## Introduction

Strain engineering has become a strong approach to improve the performance of functional materials, especially for semiconductors [1–3]. The introduction of strain into semiconductors is significant to the studies involving basic science as well as device applications. The strain-induced piezoelectric and piezoresistive effects are two crucial mechanisms in wurtzite-structured and asymmetric structure crystal. For bulk materials, the piezoelectric effect always exists in brittle materials, such as piezoelectric ceramics [4], and the piezoresistive effect has been well studied in semiconductors [5]. Interestingly, the mechanical properties of materials become more excellent when their size decreases to the nanoscale, which facilitate their application on the fabrication of flexible devices. Meanwhile, the properties of nanomaterials are sensitive to the strain due to the ultrathin size which can be used to manufacture sensors and offers method to improve their properties. More importantly, the strain tuning electric and optoelectric in low-dimensional semiconductors give rise to many high-performance and multifunctional fascinating devices which will be applied widely in semiconductor technology.

Single-atomic-layer graphene was peeled off by adhesive tape from graphite in 2004 [6]; since then, graphene-like 2D materials have been gradually brought into the limelight. In the last decade, more and more 2D crystals have been uncovered and synthesized. Recently, 1825 low-exfoliation-energy materials were selected from more than 100,000 kinds of three-dimensional compounds, containing intrinsic metallic, semi-conducting, insulating and magnetic materials [7]. 2D materials have demonstrated unparalleled opportunities in science and industry and have caused great attention due to their sub-nanometer thickness, unique structure and unusual electronic properties, which provide advance in electronic [8], optoelectronic [9–13], ultrafast photonics [14–19], sensor [20–22], energy field [23–28] and biological medicine [29–32]. Some novel physical phenomena have been found and proved in 2D materials, such as Hofstadter butterfly, Ising superconductivity, valley Hall effect and valleytronics [33–36]. Both theoretical predictions and experimental results demonstrated the excellent mechanical properties of 2D materials, such as high elasticity modulus (180 ± 60 N m^−1^ for monolayer MoS_2_) and Young’s modulus (270 ± 100 GPa for monolayer MoS_2_) and can sustain over 10% strain [37]. The good mechanical properties of 2D semiconductors provide opportunities in developing flexible electronic devices as well as conditions for studying the strain-engineered their properties.

The piezoelectric and piezoresistive effect is predicted in 2D transition metal dichalcogenides (TMDs) as early as 2012, and the two effects were observed in experiment subsequently [38–40]. Recently, the effect of strain on phonon structure, band structure, interface characteristic, transport behavior and optoelectronic performance of 2D semiconductors was investigated [2, 41, 42]. The strain effect in emerging new 2D semiconductors has made great progress in theoretical calculations. And some experimental methods were developed. Besides, the 2D semiconductors with the relatively mature synthetic technology have been studied in force sensors, piezoelectric optoelectronic devices, piezoelectric generators, etc. So far, the review article which covered both piezoelectric and piezoresistive effects caused by strain in 2D semiconductors has not been mentioned; however, both the effects have large impact on the photoelectric properties of 2D semiconductors and the synergistic effects are worth studying.

**Fig. 1:**
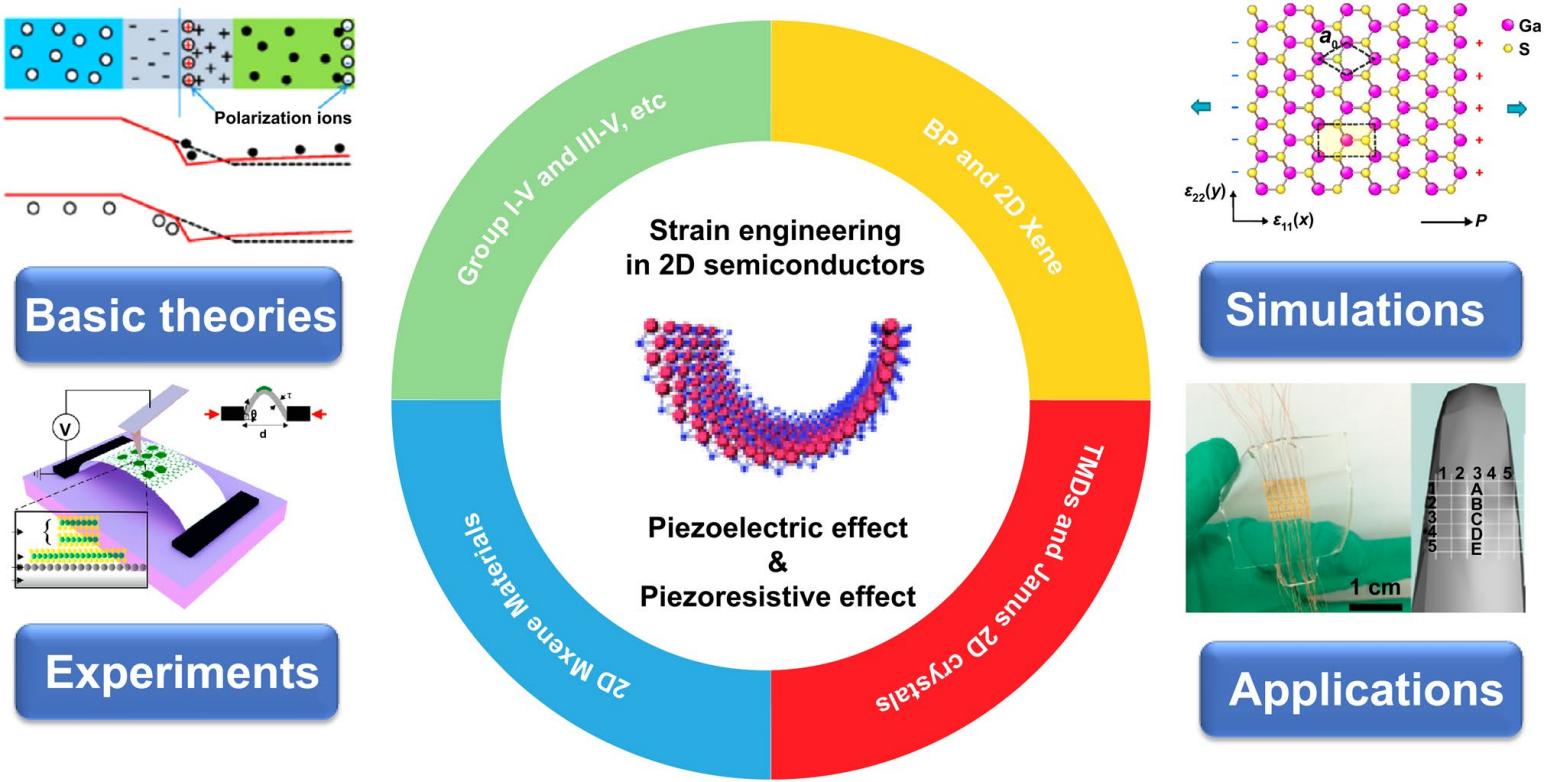
Strain engineering in 2D materials: from basic theories to applications [81, 200, 222–224]

Here, we comprehensively review the recent progress of strain-induced piezoelectric effect and piezoresistive effect in 2D semiconductors and their electronic and optoelectronic applications. As illustrated in Fig. 1, we outline the fundamental theories, simulation studies, experiments characterization and device applications and provide an outlook on the future research directions and the applications in electronics and optoelectronics of strain-engineered 2D materials. This work has significance for improving the performance and multifunctional of 2D semiconductors and developing novel devices for their applications in electronics and optoelectronics.

## Fundamental Theories and Simulations

Strain-induced piezoelectric and piezoresistive effect has been found for more than 140 [43] and 80 years [44], respectively, and there are some relatively mature theoretical bases demonstrating new opportunities in 2D materials. The broad basic concepts, commonly used mechanisms and their historical evolution of strain in materials are described in “Theories” section, and some typical calculation results of 2D semiconductors by using different methods based on the density functional theory are exhibited in “Simulation studies” section.

### Theories of Strain in Semiconductors

#### Piezoelectric Effect

The piezoelectric effect is the generation of electricity or electric polarization in a crystal as a result of applying mechanical strain. The piezoelectric effect was first detected in quartz by the brothers Jacques and Pierre Curie in 1880 [43]. Most concerned piezoelectric materials are insulated ceramics at the early stage, such as lead zirconate titanate (PZT), which have been successfully applied to commercial sensor [45]. The piezoelectric effect in nanostructured semiconductor attracts more and more attentions in recent years since Zhong Lin Wang group observed piezoelectric generation in a ZnO nanowire and coined a new term in 2006, which is piezotronics [46–48]. The piezotronics takes advantage of the coupling properties of piezoelectric effect and semiconductor characteristic. Then, the piezo-phototronics was also put forward by Zhong Lin Wang, which introduces the photoexcitation into piezotronics [49].

The piezoelectricity exists in the semiconductors whose crystal structure has non-central symmetry, for instance, wurtzite and zinc-blende crystal structures. The center of positive and negative ions is coincident without applied force, and the total dipole moment is zero. While the center of ions is not coincide when applied force and create nonzero dipole moment unit in the crystal [50]. Then, superposition of all nonzero dipole moment units forms a macroscopic potential difference along the force direction in the materials. The piezoelectric effect can be defined by the piezoelectric coefficients of *d*_*ij*_, *e*_*ij*_, *g*_*ij*_ and *h*_*ij*_, the equations of which are given as follows [51]:

2.1 $$\begin{aligned} & d_{ij} = \left( {\frac{{\partial D_{i} }}{{\partial X_{j} }}} \right)^{E} = \left( {\frac{{\partial x_{i} }}{{\partial E_{j} }}} \right)^{X} , \\ & e_{ij} = \left( {\frac{{\partial D_{i} }}{{\partial x_{j} }}} \right)^{E} = \left( {\frac{{\partial X_{i} }}{{\partial E_{j} }}} \right)^{x} , \\ & g_{ij} = \left( {\frac{{\partial E_{i} }}{{\partial X_{j} }}} \right)^{D} = \left( {\frac{{\partial x_{i} }}{{\partial D_{j} }}} \right)^{X} , \\ & h_{ij} = \left( {\frac{{\partial E_{i} }}{{\partial x_{j} }}} \right)^{D} = \left( {\frac{{\partial X_{i} }}{{\partial D_{j} }}} \right)^{x} , \\ \end{aligned}$$

where *D* is the induced electric, *E* is the electric field strength, *X* is the mechanical force and *x* is the strain. The front set of four equations corresponds to the direct piezoelectric effect, and the latter set corresponds to the converse piezoelectric effect. There are 21 non-centrosymmetric crystal classes in a total of 32 crystal classes, and 20 exhibit direct piezoelectricity. The strong piezoelectricity was observed in the group III–V and II–VI semiconductors, especially for wurtzite structure, such as GaN, InN, AlN and ZnO.

**Fig. 2:**
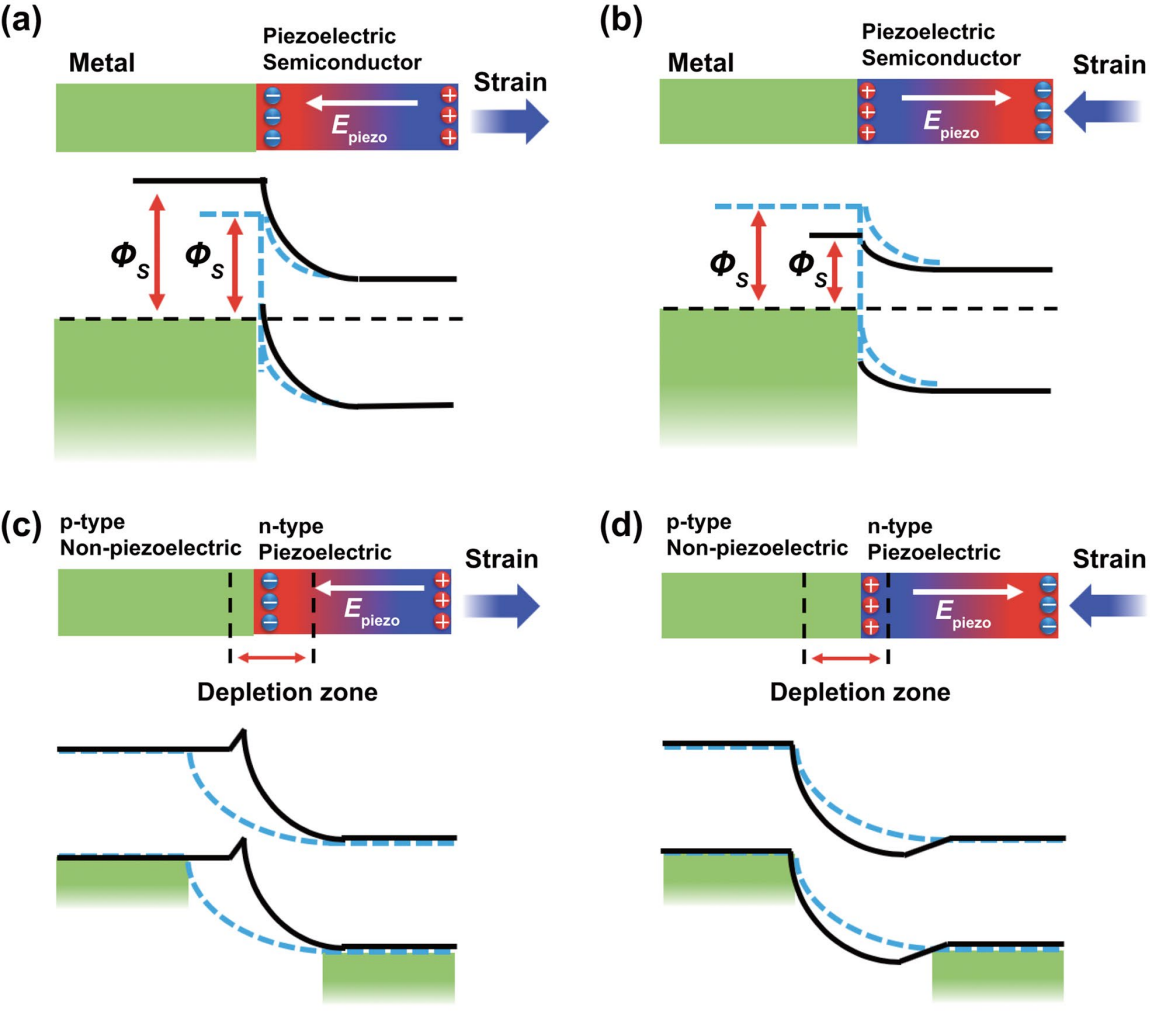
Schematic for illustrating the modulation of photoelectric device based on 2D piezoelectric semiconductors. The variation of energy-band profiles and carrier distribution of a Schottky contact induced by tensile strain (**a**) and compressive strain (**b**). The variation of energy-band profiles and carrier distribution of a *p*–*n* junction induced by tensile strain (**c**) and compressive strain (**d**)

In semiconductors, the electrons and holes in the semiconductor will move to opposite directions under the influence of the piezoelectric potential, which is named piezoelectric polarization effect [52]. The electronic transport and photoelectric properties will be modulated through the modulated interfacial properties by piezoelectric effect [53]. The performance of two typical devices based on metal–semiconductor (MS) Schottky contacts and *p*–*n* junctions could be modulated by piezoelectric potential. Taking a MS Schottky contact between *n*-type piezoelectric semiconductor and metal electrode as an example, the strain-induced electrons or holes move to the interface between metal and semiconductor. The enrichment of electrons at the interface leads to increased Schottky barrier height (SBH), as shown in Fig. 2a, while the enrichment of holes at the interface leads to decreased SBH, as shown in Fig. 2b. The principle will become complicated for *p*–*n* heterojunction, take a *p*–*n* heterojunction between a *n*-type piezoelectric semiconductor and a *p*-type semiconductor without piezoelectric as an example, and the enrichment of electrons at the interface results in that the depletion width increased in *n*-type part and decreased in *p*-type part. Meanwhile, the depletion region moves to the *n*-type semiconductor part (Fig. 2c), while the opposite happened when there is the enrichment of holes at the interface (Fig. 2d) [54]. The piezo-phototronics introduces the optical process on the basis of the coupling between piezoelectricity and electric transport. Strain-induced piezoelectric potential can effectively affect the optical processes, such as separation and recombination of photogenerated electron and holes in optoelectronic devices based on piezoelectric semiconductors [55].

#### Piezoresistive Effect

The piezoresistive effect is a change in the electrical resistivity of a semiconductor or metal when mechanical strain is applied. Different from the piezoelectric effect, the piezoresistive effect causes a change only in electrical resistance, not in electric potential. The strain-induced change in conductivity of metal was first found in 1856 [56], and the term of piezoresistive effect was first coined by Cookson in 1935 [44]. The piezoresistive effect can be observed in a metal or a semiconductor, while the piezoresistive effect in semiconductor materials is generally much stronger than in metals. The resistance (*R*) of a material is defined as: *R *= *ρl*/*a*, where *l* is the length, *a* is the cross-sectional area of the material and *ρ* is electrical resistivity. The change in *R* with force is normally a function of strain and *R*, which can be described by the gauge factor (GF) as follows:

2.2 $${\text{GF}} = \frac{{{{\Delta R} \mathord{\left/ {\vphantom {{\Delta R} R}} \right. \kern-0pt} R}}}{e} = 1 + 2\nu + \frac{\Delta \rho }{\rho }$$

where *R* is the original resistance, Δ*R* is the change in resistance, *ε* is the strain and *ν* is the Poisson’s ratio. The GF of semiconductors is two orders of magnitudes larger than that of metals which is only 0.8–3.0, while the GF of ZnO is about 350 [57]. The piezoresistance coefficient is always relative to the change in conductivity when applying force; the computational formula is similar to Eq. (2.2), as follows [45]:

2.3 $$\pi_{l}^{\sigma } = \frac{{{{\Delta \sigma } \mathord{\left/ {\vphantom {{\Delta \sigma } \sigma }} \right. \kern-0pt} \sigma }}}{X}$$

where *σ* is the original conductivity, Δ*σ* is the change in conductivity and *X* is the force. Smith et al. have learned the effect of strain on the semiconductors of silicon and germanium in 1954 [58]. The piezoresistance coefficient will increase with the decrease in scale of materials. Take silicon as an example; the piezoresistance coefficient of a Si nanowires reaches up to − 3550 × 10^−11^/Pa, whose value is − 94 × 10^−11^/Pa for a bulk material [59]. The GF of carbon nanotube can be as high as 2900 [60].

The piezoresistive effect is anisotropic for a single crystal which is associated with Miller indices of the crystal. For instance, the piezoresistance coefficient of 2D ReS_2_ is positive along *a*-axes, but negative along *b*-axes, with the ratio of GF being GFa/GFb = − 1:1.21 [61]. The state of force for a crystal can be expressed by nine components, *σ*_*ij*_ as follows:

2.4 $$\sigma = \left[ {\begin{array}{*{20}l} {\sigma_{11} } \hfill & {\sigma_{12} } \hfill & {\sigma_{13} } \hfill \\ {\sigma_{21} } \hfill & {\sigma_{22} } \hfill & {\sigma_{23} } \hfill \\ {\sigma_{31} } \hfill & {\sigma_{32} } \hfill & {\sigma_{33} } \hfill \\ \end{array} } \right]$$

where *i* indicates the direction of the applied force, while *j* denotes the direction of the force. The strain can be inferred from applied force by Hooke’s law for a homogeneous material, as follows:

2.5 $$\sigma = \varepsilon E$$

where *E* is the Young’s modulus.

The large piezoresistive effects in semiconductors demanded a fundamental theory of the physics. Strain in a crystalline solid modifies the lattice constants and reduces the crystal symmetry, leading to significant shifts in the energy band edges [62]. The existing theories were based on the change in energy band structure (including band warping and splitting) [63]. The density of states and lattice symmetry of the crystals change under the applied  strain. Then, the shift of band gap and the change in electrons mass under strain lead to the change in conductivity and carrier mobility [64]. Most theoretical models exhibit crystal orientation dependence of band structure, electron energies and the effective masses of the carriers [65].

### Simulation Studies

The 2D semiconducting TMDs, such as MoS_2_, were first used to fabricate field effect transistors in 2011 and attracted lots of research interests because they possess a band gap [66]. The excellent mechanical properties of 2D TMDs, which fracture strains as high as 11% [67], make them promising candidates for studying the properties of strain, electric, photon and other properties coupling. A lot of papers reported the theoretical simulation of piezoelectric and piezoresistive effects in 2D TMDs materials stating in 2012. As more and more 2D materials are developed, the simulation studies of strain in these materials are always published before experimental studies.

#### Simulation Studies of Piezoelectric Effect in 2D Semiconductors

The piezoelectric properties of 2D semiconductor can be calculated by using different methods on the basis of the density functional theory (DFT), such as the norm-conserving pseudopotential [68] or projector-augmented wave potential [69] approaches. In 2017, Cheon et al. selected 325 potential 2D piezoelectric monolayers from over 50,000 inorganic crystals, and the 2D monolayers are lack of centrosymmetry and have a nonzero band gap, which are not piezoelectric in their bulk structure [70].

Based on the modern theory of polarization, the linear piezoelectric effect (*d*_*ijk*_) of a flat 2D material can be calculated by evaluating the change in polarization under uniaxial strains (*e*_*ijk*_). The third-rank piezoelectric tensors *e*_*ijk*_ and *d*_*ijk*_ can be evaluated by their respective Maxwell relations [71]:

2.6 $$\begin{aligned} e_{ijk} = \frac{{dP_{i} }}{{d\varepsilon_{jk} }} \hfill \\ d_{ijk} = \frac{{dP_{i} }}{{d\sigma_{jk} }} \hfill \\ \end{aligned}$$

where *ε*_*jk*_, *σ*_*jk*_ and *P*_*i*_ represent the strain tensor, stress tensor and polarization tensor, respectively. In the contracted Voigt notation, the *e*_*ijk*_ and *d*_*ijk*_ are reduced to *e*_il_ and *d*_il_, respectively. The DFT simulations are used to calculate the piezoelectric coefficients of *e*_il_ and *d*_il_ using the relation:

2.7 $$e_{il} = d_{ik} C_{kl}$$

where *C*_*kl*_ is the elastic stiffness tensor. The *e*_*il*_, *d*_*il*_ and *C*_*kl*_ tensor is restricted by the symmetry elements of a crystal, and the number of independent tensor coefficients can be further reduced using the point groups of the 2D materials. Take 2H phase TMDs as example, which belong to the *D*_3*h*_ point group symmetry and the in-plane and out-of-plane piezoelectric coefficients of *e*_11_, *d*_11_, *e*_31_ and *d*_31_, whose values are related to the elastic stiffness coefficients as:

2.8 $$\begin{aligned} d_{11} = \frac{{e_{11} }}{{C_{11} - C_{12} }} \hfill \\ d_{31} = \frac{{e_{31} }}{{C_{11} + C_{12} }} \hfill \\ \end{aligned}$$

For the 2D materials with *C*_2*v*_ point group symmetry, the piezoelectric coefficients can be described as [72]:

2.9 $$\begin{aligned} d_{11} = \frac{{e_{11} C_{22} - e_{12} C_{12} }}{{C_{11} C_{12} - C_{12}^{2} }} \hfill \\ d_{12} = \frac{{e_{12} C_{11} - e_{11} C_{12} }}{{C_{11} C_{12} - C_{12}^{2} }} \hfill \\ \end{aligned}$$

**Fig. 3:**
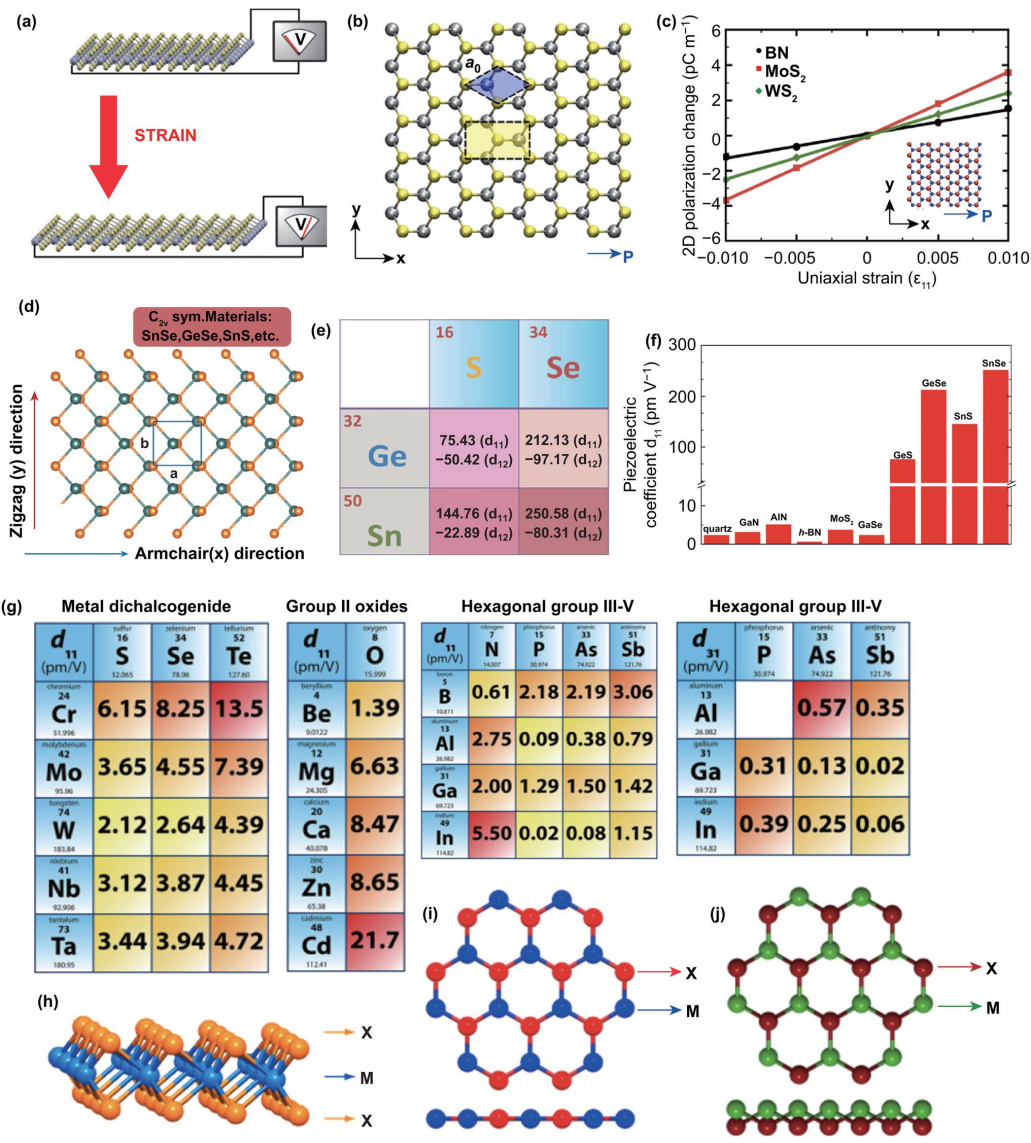
Simulation studies of in-plane piezoelectric effects in 2D semiconductors. **a** Schematic diagram of piezoelectric potential generation in monolayer materials. **b** Monolayer top-view geometry of monolayer 2H-MoS_2_. **c** Variation of polarization induced by strain in monolayer materials. Reproduced with permission [73]. Copyright 2012, American Chemical Society. **d** Top views of the C_2v_ orthorhombic monolayer. **e** Calculated piezoelectric coefficients of monolayer group IV monochalcogenides and **f** comparison of piezoelectric coefficient (*d*_11_) with previously known piezoelectric materials. Reproduced with permission. [77] Copyright 2013, American Chemical Society. **g** Calculated piezoelectric coefficients of 2D materials. Materials structures are illustrated for **h** 2H, **i** planar hexagonal and **j** buckled hexagonal structures. Reproduced with permission. [91] Copyright 2015, American Chemical Society

At present, people pay more attention to the in-plane piezoelectric effect of 2D semiconductors, especially to the piezoelectric effect in the armchair crystal direction of monolayer TMDs. In 2012, Duerloo et al. [73] have predicted the piezoelectric in 2D TMDs for the first time. Figure 3a, b shows the schematic diagram of piezoelectric potential generation in monolayer materials. The piezoelectric coefficients were calculated by using the generalized gradient approximation based on DFT. TMDs are a class of layered materials with the formula MX_2_, where M is a transition metal element from group IV, group V or group VI and X is the chalcogen [74]. Monolayer TMDs with trigonal prismatic (*D*_3h_) and octahedral (*D*_3d_) coordinations correspond to 2H and 1T phase. The 2H-phase TMDs usually behave as semiconductors, while the 1T-phase TMDs are metallic [75]. The top view of 2H TMDs in Fig. 3b shows a honeycomb structure where adjacent sites are occupied by two alternating species. Each layer formed X–M–X structure with the chalcogen atoms in two hexagonal planes separated by a plane of metal atoms, as shown in Fig. 3h. The strain dependence of piezoelectric polarization is shown in Fig. 3c. The results show a periodic trend of *e*_11_ and *d*_11_ coefficients in the 2D TMDs monolayers. The symmetry of monolayer hexagonal structures TMDs belongs to *D*_3h_ group, whose symmetry is broken and gives rise to piezoelectricity.

Fei et al. [76] found the large piezoelectricity in monolayer group IV monochalcogenides, such as monolayer SnS, SnSe, GeS and GeSe, and their atomic structure exhibited a *C*_2v_ point group, as presented in Fig. 3d. Their monolayer structures are non-centrosymmetric allowing them to be piezoelectric. The band gaps of monolayer MX are between 1.2 and 2.7 eV, with huge excitonic effects, which are promising for solar energy applications [77]. Figure 3e, f shows that the piezoelectric coefficients (*e*_11_, *e*_12_, *d*_11_, and *d*_12_) of monolayer MX are 10–100 times larger than conventionally used piezoelectric semiconductors, such as ZnO and monolayer MoS_2_. They also found that the *d*_11_ and *d*_12_ coefficients follow a periodic trend, monolayer GeS possesses the minimum value of piezoelectric coefficient and monolayer SnSe has the largest coefficient in which d_11_ is up to 250.58 pm V^−1^. More detailed theoretical calculations of piezoelectric coefficient of two stable structural phases (A-MX and H-MX phases) of monolayer group IV monochalcogenides have been reported by Ting et al. [72]. The piezoelectric coefficient of A-MX phase monolayers is 1–2 magnitude orders larger than that of H-MX phase monolayers. The similar results of A-MX and H-MX phase are also reported in any other paper [78, 79]. Recently, the performance of 2D piezophototronic in monolayer group IV monochalcogenides has been calculated by Michael et al., and the effect of piezoelectric potential on MS Schottky barrier contact was investigated [80]. The output power, open-circuit voltage, fill factor and power conversion efficiency of the monolayer MX-based solar cell are decreasing with the applied strain increasing from − 1 to 1%. It indicates that the compressive strain helps to improve the photoelectric properties of the IV monochalcogenides monolayers.

The group III–V buckled honeycomb monolayers belong to the point group of 3 m and have no inversion symmetry. The 3-m symmetry of these bucked structures supports nonzero *e*_11_, *e*_31_, *d*_11_, and *d*_31_ piezoelectric coefficients. The piezoelectric effect in monolayer GaS, GaSe and InSe was also calculated by using first-principle calculations, and those materials have linear ε_11_ piezoelectric coefficients and the same order of magnitude as monolayer MoS_2_ and h-BN [81]. The high piezoelectric effects have also been predicated in monolayer SbAs, SbN and SbP, whose calculated *d*_11_ piezoelectric coefficients are 243.45, 142.44, and 118.29 pm V^−1^, respectively [82].

Black phosphorus (BP) is a layered material where all layers are stacked together by weak van der Waals interactions [83]. Similar to graphene, each phosphorus atom is bound to three neighbors. However, unlike graphene, BP displays out-of-plane distortion, resulting in a ridge structure along the zigzag direction and a puckered structure along the armchair direction, and thus non-equal bond lengths and bond angles [84]. This leads to BP with a linked ring structure and substantial in-plane anisotropy including optical, electrical, thermal and mechanical anisotropy [85, 86]. Due to its non-centrosymmetric crystal structure, the piezoelectric property of BP has been demonstrated. Drissi et al. [87] used DFT calculations to predict the piezoelectric responses of BP. The calculations show the values of force piezoelectric responses values: *e*_11_ is 59 pC m^−1^ and *e*_31_ is 1.06 pC m^−1^. The calculated piezoelectric coefficient *d*_11_ *is* − 9.48 pm V^−1^, which is comparable with other known 2D metal dichalcogenides. The chemical functionalization with oxygen atoms or surface oxidation can break the structural symmetry of BP and enhanced its piezoelectric properties. Piezoelectric effects of surface-oxidized BP were studied by using DFT method by Li et al. [88]. The piezoelectric coefficient *d*_11_ for surface-oxidized BP is up to 88.54 pm V^−1^, which is larger than that of 2D h-BN and MoS_2_. The piezoelectric effect of 2D materials can also be applied to tune the properties of *p*–*n* heterostructure. Huang et al. [89] investigated the electronic transport and optical properties of BP/MoS_2_ bilayer by first-principle calculations. The results show that the band gap of BP/MoS_2_ heterostructures decreases with the increase in applied compressive strain and found a semiconductor to metal transition. The results also show that the carrier effective mass and carrier concentration of BP/MoS_2_ junctions can also be controlled by the applied strain. Moreover, the band alignment of BP/MoS_2_ bilayer can be tunable under applied compressive strain, which can facilitate carriers transferring between BP and MoS_2_ layer. The first-principle simulation results show that piezoelectricity of 2D monolayer phosphorene oxides is enhanced, with piezoelectric coefficient *d*_11_, *d*_31_ and *d*_26_ with values of 54, 10 and 21 pm V^−1^, respectively [90].

More systematic study has been reported by Blonsky and his colleagues [91]. The calculated results show that the monolayer WS_2_ has the smallest piezoelectric coefficient, the largest coefficient was found in monolayer CrTe_2_ (Fig. 3g). The calculated piezoelectric coefficients of monolayer MoS_2_ are compared favorably with bulk α-quartz and wurtzite GaN, and the piezoelectric coefficients of CrTe_2_ are much larger than frequently used piezoelectric semiconductor.

**Fig. 4:**
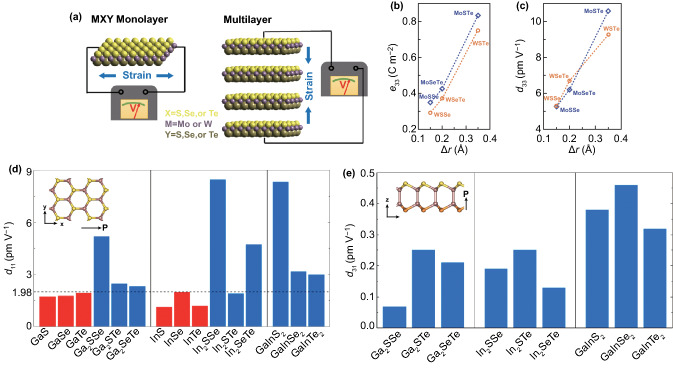
Simulation studies of out-of-plane piezoelectric effect in 2D semiconductors. **a** Schematic diagram of in-plane and out-of-plane piezoelectric potential generation in Janus monolayer TMDs. The out-of-plane piezoelectric coefficient **b** *e*_33_ and **c** *d*_33_ of multilayer Janus monolayer TMDs. Reproduced with permission [95]. Copyright 2017, American Chemical Society. The piezoelectric coefficients **d** *d*_11_ and **e** *d*_31_ of Janus group III chalcogenide monolayers; the inset is top view and side view of Janus structures M_2_XX’. Reproduced with permission [97]. Copyright 2017, AIP Publishing

Few people pay attentions to the out-of-plane piezoelectric properties of monolayer crystals early due to their vertically symmetrical. Recently, Janus 2D semiconductors have become a research hotspot thanks to their out-of-plane asymmetry structure and display piezoelectric effect [92, 93]. Different from conventional 2D materials which only have in-plane piezoelectricity, the Janus 2D materials possess both out-of-plane and in-plane piezoelectricity. Janus 2D TMD materials were first synthesized in 2017, and their piezoelectricity was calculated then [94]. Dong et al. reported the out-of-plane and in-plane piezoelectric effect in Janus 2D TMDs based on ab initio calculations [95]. The Janus MXY monolayers (such as MoSSe) are similar to 2H MX_2_ monolayers (such as MoS_2_). In comparison with regular MX_x_Y_1-x_, the X atoms and Y atoms in Janus monolayers are never coplanar. The crystal structure and schematic diagram of out-of-plane and in-plane piezoelectric potential generation in Janus 2D TMDs are shown in Fig. 4a. The un-equivalent of M–X and M–Y bonding lengths in MXY monolayers leads to the out-of-plane piezoelectricity. The results show that the in-plane piezoelectric coefficient (*e*_11_ and *d*_11_) of monolayer MXY is fall between the values of MX_2_ and MY_2_, and the *d*_11_ value is between 2.02 and 7.00 pm V^−1^. The out-of-plane piezoelectric coefficient (*e*_31_ and *d*_31_) of monolayer MXY is smaller than the in-plane values, whose values are only between 0.007 and 0.038 pm V^−1^. More significantly, the piezoelectric coefficient of some MXY multilayers is enhanced, the *e*_31_ values of WSTe increase from 0.010 to 0.140 C m^−2^ (Fig. 4b), and the *d*_31_ value of MoSTe increases from 0.030 to 0.447 pm V^−1^ (Fig. 4c). DFT's calculated results show that the piezoelectric coefficient *d*_22_ of the Janus semiconducting group IVB TMDs is 4.68–14.58 pm V^−1^, and the max d_31_ value is 0.414 pm V^−1^. The results also indicate that the applied 9% strain along the armchair direction can dramatically enhance the *d*_22_ values increasing from 4.68 to 123.04 pm V^−1^ for HfSSe Janus monolayers [96]. There are two kinds of Janus structures in group III monochalcogenide monolayers due to their four-atomic-layer structure, which is M_2_XX’ and MM’X_2_. The results show that the in-plane piezoelectric coefficient *d*_11_ of Janus monolayers is higher than that of conventional monolayers (Fig. 4d). The out-of-plane piezoelectric coefficients *d*_31_ are 0.07–0.46 pm V^−1^ (Fig. 4e) [97]. Cai et al. found that the in-plane interlayer sliding in bilayer Janus monolayer TMDs could enhance the out-of-plane piezoelectric effect [98]. Overall, the Janus 2D materials with both in-plane and out-of-plane piezoelectric effects offer a new sight for 2Dsemiconductros in piezoelectric-related applications [99].

Some advantages of 2D piezoelectric materials can be inferred from this part. First of all, some non-piezoelectric bulk materials will have intrinsic piezoelectric characteristics in their 2D crystals due to the broken inversion symmetry. And the relatively higher piezoelectric coefficient was calculated in 2D semiconductors, such as monolayer group IV monochalcogenides. In addition, the piezoelectric properties of 2D materials can be modulated through structural regulation and surface modification, while the piezoelectricity in 2D TMDs is limited to in-plane specific direction and odd number layers, which makes it to be limited in application. As more and more 2D piezoelectric semiconductors are discovered, such as wurtzite structure, Janus materials and monolayer SnSe have been predicted to process highest piezoelectric coefficient. Their piezoelectric properties urgently need to be observed experimentally.

#### Simulation Studies of Piezoresistive Effect in 2D Semiconductors

Different from the simulations of piezoelectric coefficients, the researchers rarely calculate changes in resistivity directly in 2D materials by using first-principle calculations. It is usually predicted by calculating the energy band and effective carrier mass. The change in lattice constant under strain is the basis for calculating band structure of strained materials by using DFT. The theoretical mechanical strains can be calculated by *ε *= Δ*a*/*a*_0_, where *a*_0_ is the lattice constant without strain and Δ*a* is the change in the lattice constant under strain [100]. There is the change in variation of the electronic band structure of monolayer TMDs with the applied five different types of strain, such as tensile and compression strain [38]. The results show that monolayer TMDs are very sensitive to the strain. Particularly, it is found that the tensile and shear strain can increase the valence band and decrease conduction band, causing the reduction in TMDs’ band gap. The band gap of 2D TMDs reduced faster under biaxial strain than that under uniaxial strain, and the monolayer semiconductors will turn into metals under over 10% strain, which stems from the overlapping of *d*_z_^2^ orbital at Fermi level. In addition, a direct-to-indirect band gap is observed in 2D TMDs under 1–3% tensile strain, and the needed strain is gradually increased in chalcogens from sulfides to tellurides, whose strain for MoS_2_ is about 1% and for MoSe_2_ up to 3% [101]. The band gap of monolayer MTe_2_ decreases relatively faster with increasing strain, and a semiconductor-to-metal transition needs 10% strain due to the diffuse nature of heavier chalcogens [102]. Some similar results were also published during the same period [103–105].

Deformation potential (DP) is the variation of the electronic band structure with applied strain. The effect of strain on DP and effective masses of electrons and holes has also been reported [106]. The simulations results reported by Chang et al. demonstrated that the band gaps of 2D TMDs most depend on the X–X bond length and the X–M–X bond angle, and the direct band gaps can be obviously widened by applied compressive biaxial strains [107, 108]. Lu’s results also showed that the direct band gap can be enlarged at first under the compressive strain less than 2% [109, 110].

Bilayer TMDs are usually indirect band gap semiconductors. Similar to monolayer TMDs, the in-plane strains can also reduce band gap energy of bilayer TMDs and cause semiconductor–metal transitions when applying a critical strain. The change rule of energy band structure depends on the types of applied strain [111]. The carrier effective masses in bilayer MoS_2_ can also be tuned by applied strain [112].

**Fig. 5:**
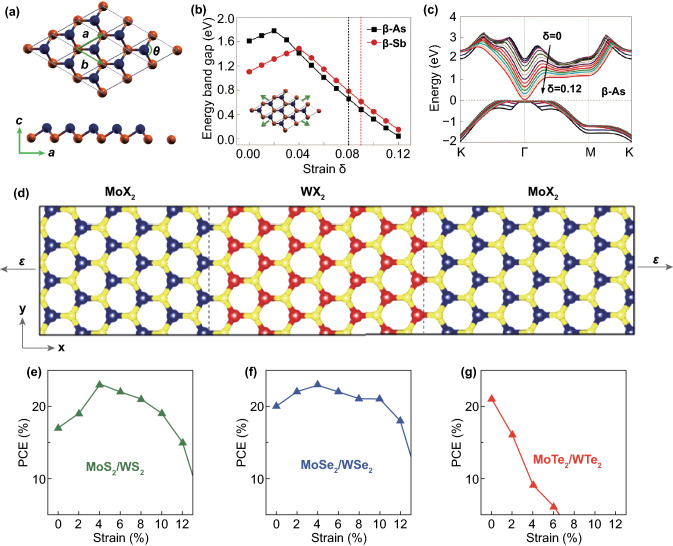
Simulation studies of piezoresistive effect in 2D semiconductors and their heterostructures. **a** Crystal structures of monolayer β-As and β-Sb. **b** Band gap variation under biaxial tensile strain. The dot dashed line shows the position of the critical strain of indirect-direct gap transition. **c** Positions of conduction and valence band edges under different strains. Reproduced with permission [114, 115]. Copyright 2018, Royal Society of Chemistry. **d** Geometric structures of the heterostructures containing a total of twenty MoX_2_ and WX_2_ units per supercell and applied uniaxial strain ε along the armchair direction. **e–g** Strain dependence of PCE in lateral heterostructures. Reproduced with permission [118]. Copyright 2017, IOP Publishing

The 2D wide band gap semiconductors arsenene and antimonene of β-phase (which is most stable) were developed based on first-principle calculations in 2015 [113]. Figure 5a shows the crystal structures (top and side views) of arsenene. The single-layer arsenene and antimonene are indirect band gap semiconductor which is not conducive to its application in optoelectronic. Interestingly, the results show that the band gap is decreased with the increase in biaxial strain and a significant indirect-to-direct band gap transition in arsenene and antimonene occurs when applying a relatively small strain. The CBM moves to G high symmetry point, which implies the arsenene turns into a direct band gap semiconductor. As shown in Fig. 5b, with applied strain increasing, the band gap of arsenene and antimonene increases initially and decreases afterward. Indirect-to-direct band gap transition occurs when applying 8% and 9% for arsenene and antimonene. Figure 5c shows the variations of band structure of arsenene under different strains [114]. The direct band of the strained monolayers arsenene and antimonene has obvious advantages for their applications in optoelectronic devices. Subsequently, the effect of strain on the electrical transport and photoelectric properties of single-layer arsenene and antimonene was also predicted by DFT. Simulation results of Kripalani and his colleagues show that the band gap of antimonene and arsenene is not sensitive to the applied strain along the zigzag direction of the crystals, while an indirect–direct band gap transformation is observed when applied 4% strain along the armchair directions [115]. And the work function of suspended antimonene increases from 4.59 to 5.07 eV under applied 4% biaxial strain [115]. Shu et al. [114] employed DFT combined with G_0_W_0_, and BSE calculations also found that the optical absorption spectra of monolayer arsenene and antimonene redshift significantly with increasing strain and the optical absorption is improved in the range of 1.2–2.2 eV energy region. In addition, they also found that the optical absorptions can be enhanced by strong electric field.

Monolayer SnSe is a nearly direct band gap semiconductor. The direct–indirect band gap transition caused by applied strains was also found in monolayer SnSe. And the band gap reduces from 1.05 to 0 eV when applying 12% compression strain, while the band gap only decreases to about 0.7 eV applied 12% tensile strain. The band gap of the sample under 12% tension biaxial strain is always higher than that of under 12% compression strain, and 2–4% compression can enlarge the band gap of SnSe [79]. DFT calculations show that the light absorption of monolayer SnSe is increased in the ultraviolet region by applied strain [116]. The electron and hole mobility are decreased with the increase in biaxial tensile strain, which is due to the increasing effective mass of carriers caused by strain, in which electrons increased by 147% and holes increased by 968% under a small biaxial tensile strain [117].

Lee et al. [118] used first-principle DFT calculations, which show that the uniaxial tensile strain can be used to improve optical properties of 2D TMDs lateral heterostructures MoX_2_-WX_2_ (X = S, Se, Te). A uniaxial strain is applied along the armchair direction of the lateral heterostructures, as shown in Fig. 5d. The applied strain can significantly improve the power conversion efficiency (PCE) of MoX_2_-WX_2_ lateral heterostructures due to the strain-induced band gap offset. Under 4% uniaxial tensile strain, the PCE of the MoS_2_/WS_2_ (Fig. 5e) and MoSe_2_/WSe_2_ (Fig. 5f) heterostructures can be increased by about 35% and 15% compared with that of the unstrained system, while the PCE of lateral MoTe_2_/WTe_2_ (Fig. 5g) heterostructures is continuously decreased under the uniaxial strains. The effect of intrinsic strain (induced by the lattice mismatch) on the properties of in-plane junctions based on 2D TMDs was also calculated. The intrinsic strain in MoSe_2_/WSe_2_ and MoS_2_/WS_2_ interface can be ignored due to the small lattice mismatch. However, the intrinsic strain in MoS_2_/WSe_2_ and MoS_2_/MoSe_2_ junctions can lower the coupling strength between nonmetal *p* and metal d orbitals and therefore modifies the splitting between bonding and antibonding states at the high-symmetry k-points [119]. The effect of applied strain on the electric and optoelectric performance of van der Waals heterostructures of MoS_2_/WSe_2_ [120], InSe/arsenene [121], arsenene/MoTe_2_ [122], arsenene/C_3_N [123] and MXene/blue phosphorene [124] also has been calculated.

The piezoresistive effect exists in all 2D semiconductors and is not exemplified herein. Its influence on the electrical and optoelectronic properties of 2D semiconductors cannot be ignored. The main commercial application of piezoresistive effect in materials is strain sensor. Generally, the piezoresistance coefficient, which determines the sensitivity and GF of a strain sensor, is increased with decreasing the size of materials. The wide strain controllable of band gap in 2D semiconductors suggests wide strain sensor applications, such as tactile sensor at the joint. In addition, some simulation results can support that photoelectric performance can be tuned by piezoresistive effect. Firstly, strain modulate band gap implies the change in absorption wavelength. And the strain-induced interchange between direct and indirect band gaps is associated with the efficiency of photogenerated carrier transition. In addition, the stain induced the shift of optical absorption spectra which directly determines the optical detection performance. Furthermore, under strain, the type-I band alignment can be transformed to type-II band alignment for some 2D van der Waals heterostructures, which affect light detection mechanism of the heterojunction. At last, the strain can enhance the fill factor and PCE of photovoltaic devices based on 2D heterostructure due to the piezo-potential which can further enhance the maximum output power and open circuit voltage. Therefore, the piezoresistive effect in 2D semiconductors has great application prospects in the field of high-sensitivity sensor devices and improving their photoelectric performance.

In this section, the theories of strain-engineered 2D materials are described. And we reviewed the recently simulation studies of piezoresistive effect and strain-engineered band structure in 2D semiconductors. For the calculations of piezoelectric effect, most of DFT studies were performed by the Vienna ab initio Simulation Package (VASP), and a few studies were implemented in Quantum-ESPRESSO software package. The structural relaxation and the calculations of the elastic and piezoelectric tensors usually employ the Perdew–Burke–Ernzerhof (PBE) generalized gradient approximations (GGA) exchange–correlation functional. While the piezoresistive effect of 2D semiconductor is not directly calculated, it is usually predicted by calculating the energy band and effective carrier mass. DFT calculated the change in band gap depending on the change in the crystal lattice under strain. VASP is still the most commonly used software, as well as Quantum-ESPRESSO, Cambridge Sequential Total Energy Package (CASTEP), Grid-based projector-augmented wave (GPAW) and Abinit. Electronic band structures can be calculated by a hybrid functional based on Heyd–Scuseria–Ernzerhof (HSE06). The GW approximation was treated to obtain quasiparticle (QP) energies, and the coupled electron–hole excitation energies and exciton wave functions were obtained by solving the Bethe–Salpeter equation (BSE). The calculated band gap of 2D materials based on GW is always larger than that based on DFT due to the reduced electronic screening in the 2D system [125, 126]. In order to calculate accurate band structure, the Spin–orbit coupling and van der Waals force can also be considered in calculation methods, especially for heavy elements [115]. Some simulation results have been well verified in experiment, and some significant results still need to be verified experimentally. Above all, these simulation studies point out some directions for experimental research. The vertical piezoelectric effect in 2D wurtzite crystals and Janus 2D materials seems more easier to apply to energy conversion devices and strain sensors. The piezoresistive effect in 2D b-As, b-Sb and van der Waals heterostructures needs to be confirmed which have great application prospects in optoelectronic devices.

## Experimental Studies of Strain-Engineered 2D Semiconductors

The key problem of experimentally observe piezoelectric effect and piezoresistive effect in 2D semiconductor materials is how to apply strain or force to 2D materials. So far, the strain and force can be applied by a variety of approaches. Experimental techniques to applied strain on 2D materials such as bending and stretching of flexible devices and using atomic force microscopy (AFM) tip have been summarized. When applying strain, the detection of electrical or optical signals is used to analyze the piezoelectric effect and piezoresistive effect is also very important.

### Applying Strain by Flexible Devices

**Fig. 6:**
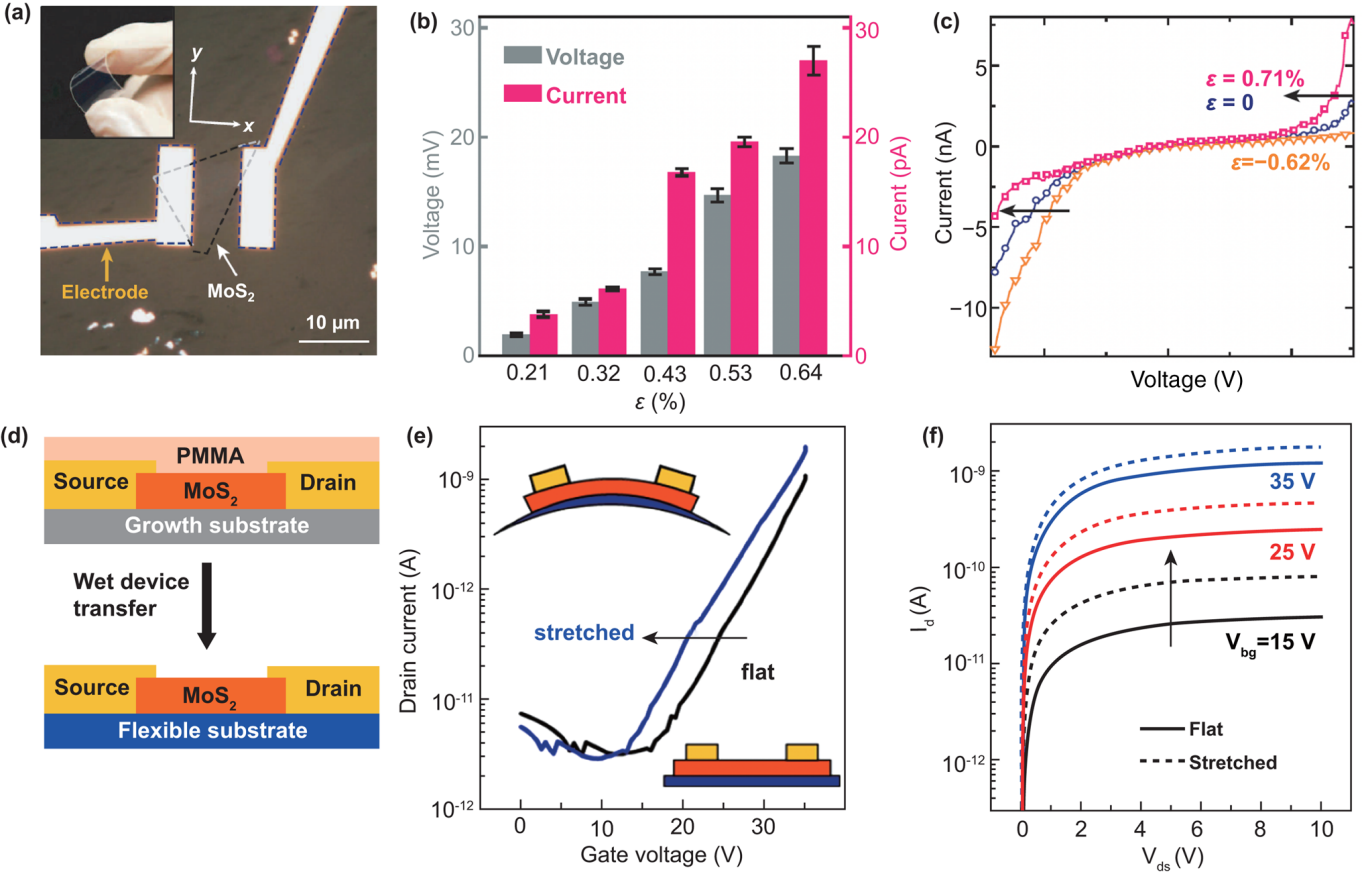
Applying strain on 2D semiconductors by flexible devices. **a** A flexible device with monolayer MoS_2_ flake along armchair direction. **b** Evolution of the piezoelectric outputs with increasing number of atomic layers in MoS_2_ flakes. **c** Asymmetric modulation of *I*–*V* curve by strains shows the piezoelectric effect. Reproduced with permission [39]. Copyright 2014, Nature Publishing Group. **d** Schematic diagram of flexible device and fabrication process. **e** Transfer curves and **f** *I*–*V* curves of a flexible monolayer MoS_2_ transistor measured before applying strain and applying 0.07% strain. Reproduced with permission [127]. Copyright 2015, American Chemical Society

Transfer samples onto a flexible substrate and applied strain through bent or elongate the substrate is one of most common methods to study the effect of strain on the performance of low dimensional materials. In 2014, the piezoelectric effect in the 2D MoS_2_ was observed experimentally for the first time by Wu et al. [39]. According to the predication, the piezoelectric effect in MoS_2_ is only in Armchair direction, so the crystal structure is confirmed by using second-harmonic generation instrument. Figure 6a shows that a 2D MoS_2_ device is fabricated on a flexible polyethylene terephthalate (PET) substrate and the strain was applied by bending the device. The piezoelectric current and voltage are increased with the increase in strain (Fig. 6b), and the variation of the piezoelectric effect with increasing the number of layers was investigated. The asymmetric change in *I*–*V* curve in a monolayer MoS_2_ device under strain in Fig. 6c shows characteristics of the piezoelectric effect. This work established the experimental basis for the 2D hexagonal piezoelectric effect, and the above conclusions are universal in the 2D hexagonal piezoelectric system. The authors also put forward some forward-looking views. They think mechanical strain can be used as a gating signal to modulate the transport behavior based on 2D material electronic devices. The piezoelectric polarization charge generated by the piezoelectricity can adjust a MS Schottky barrier or a *p*–*n* junction characteristic based on 2D semiconductors, thereby adjusting the photoelectric performance of the device.

The piezoresistive effect is a change in the electrical resistivity of a semiconductor or metal when mechanical strain is applied. The piezoresistive effect was first investigated in a flexible MoS_2_ field-effect transistors (Fig. 6d) through transport behavior in 2015 by Tsai et al. [127], which was carried out on a 2-nm-thickness (trilayer) MoS_2_. The MoS_2_ transistor is very sensitive to strain; a shift to lower back-gate voltages of transfer curve (Fig. 6e) and an increase in device current (Fig. 6f) are observed under strain. The reflection spectroscopy was used to prove the observed phenomenon originating from the change in band gap. The band gap of the trilayer MoS_2_ liner decreases from 1.58 to 1.52 eV under 0.2% strain, with a slope of − 300 meV/% strain applied. Some studies show that the small strain has little influence on the transport behavior of 2D MoS_2_ [128, 129].

**Fig. 7:**
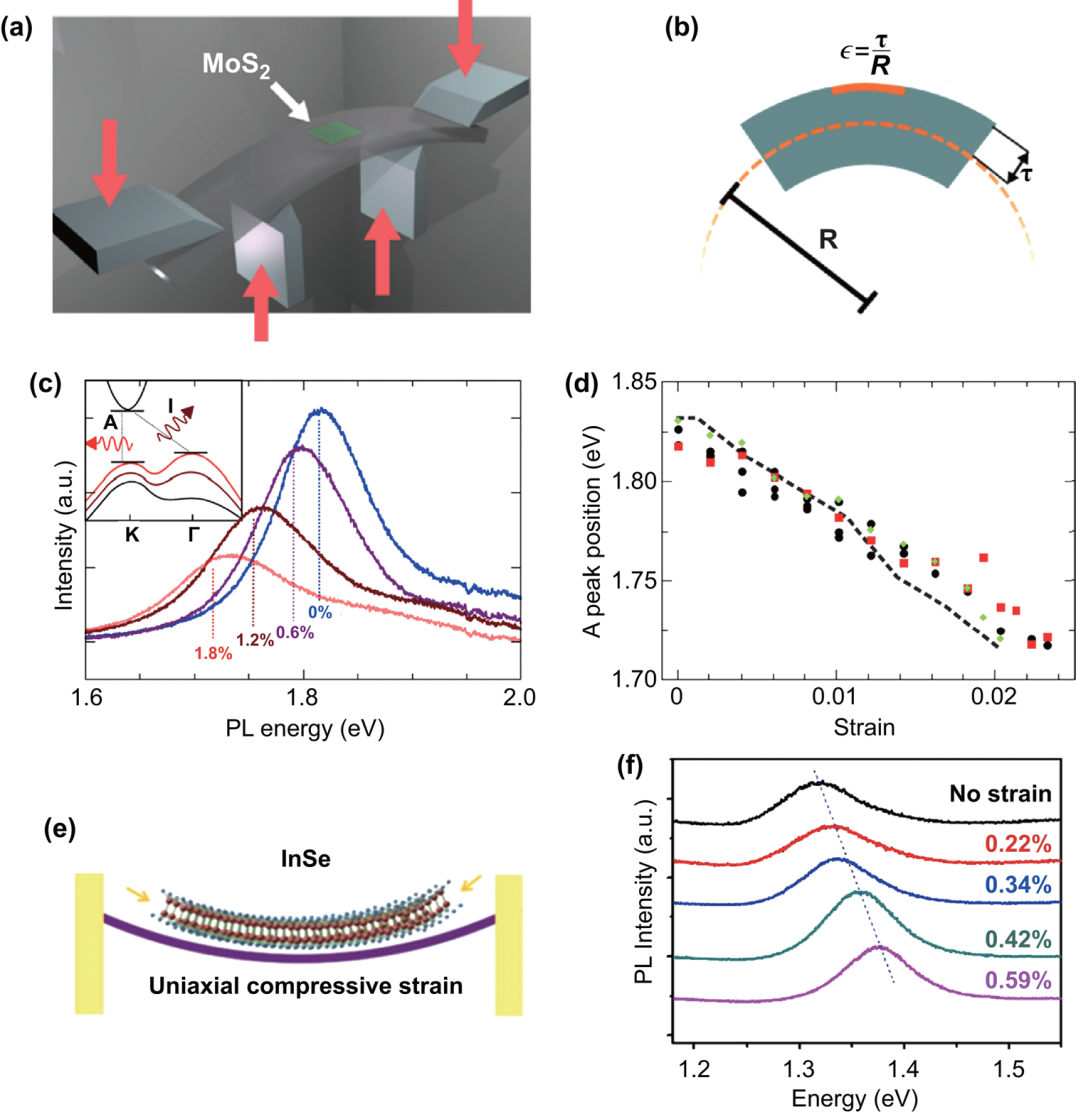
Applied uniaxial strain to 2D semiconductors. **a** Schematic of applied bending strain on MoS_2_ device and **b** the strain calculation method. **c** PL spectra of a representative monolayer device as it is strained from 0 to 1.8%. **d** Position of the PL peak as a function of strain. Reproduced with permission [40]. Copyright 2013, American Chemical Society. **e** A schematic of a two-point bending apparatus used for applying uniaxial compressive strain. **f** PL spectra of a multilayer InSe flake of thickness ~ 14 nm under different uniaxial compressive strains. Reproduced with permission [142]. Copyright 2018, IOP Publishing

Applied bending strain on a 2D graphene material was reported as early as 2009 [130], and the same method was performed on 2D MoS_2_ which was reported in 2013 [40]. The changes in the structure and properties of materials can be easily observed through the spectrum [131]. A four-point bending apparatus was used to apply uniaxial tensile strain on 2D MoS_2_, the schematic and the strain calculation method as shown in Fig. 7a, b. The applied strain is in the range of 0–2.2%. Figure 7c, d shows that the photoluminescence (PL) energy and intensity are decreased with increasing applied bending strain, revealing that a band gap decrease in monolayer and bilayer MoS_2_ is approximately ∼ 45 and ∼ 120 meV/% strain, respectively. The suppression and enhancement of PL efficiency of monolayer MoS_2_ under strain can be used for quantifying the changes in carrier populations and band structure. Besides, the direct and indirect transitions can also be identified by PL spectra.

The bending strain-induced large response in resistance of a 20-nm BP-based flexible FET was observed, while the strain has no effect on the mobility of the device. And the infrared extinction spectra results show 99 ± 4 meV/% strain in the armchair direction and 109 ± 2 meV/% strain in the zigzag direction [132]. The recent results of strain-induced band gap tuning of 2D SnSSe indicated an improved sensitivity with a decrease in the thickness of sample [133]. The method of  applied uniaxial tensile strain by transfer 2D materials onto a flexible substrate and observed the piezoresistive effect through PL spectra also have been reported in Refs. [134–141]. 

The above works are applied uniaxial tensile on 2D semiconductors; the effect of uniaxial compressive strain on the PL spectra of 2D materials has also been studied. When the 2D material adhere to outer surface of bent substrate, the uniaxial tensile strain is applied on it, Fig. 7a is an example. While when the 2D materials adhere to inner surface of bent substrate, the uniaxial compressive strain is applied on it, Fig. 7e is an example. Figure 7f shows that the band gap of InSe is increased with increasing uniaxial compressive strain, compared with a decrease in the band gap achieved under uniaxial tensile strain [142].

In most 2D materials, the tensile strain causes redshift (band gap narrowing) of its excitation spectrum and the compressive strain causes blueshift (band gap widening) of its excitation spectrum, but 2D BP is an exception. Jia et al. [143] found that the optical absorption behaviors are strain dependent, and the first absorption peak of BP monotonically blueshifts with the increasing tensile strain applied along the armchair direction, while this uncovered for that along the zigzag direction of BP. And Zhang et al. also observed similar results on a 6L BP through infrared extinction spectra [141]. The varying tensile strains (up to 0.92%) were applied along the armchair and zigzag directions of BP crystal. The results show that the two characteristic peaks of *E*_11_ and *E*_22_ both blueshift monotonically with the increase in strain. Corresponding optical band gap (*E*_11_) increases from 0.54 to 0.65 eV. The blueshift rate of *E*_11_ in armchair direction and zigzag direction is 117 and 124 meV/%, respectively. Furthermore, there is no difference in the shift of *E*_22_ in two crystal directions, which is blueshift of 99 meV/%. The tensile strain will induce decreasing distance between layers in BP due to large out-of-plane Poisson’s ratio, which will increase the interlayer interaction and band gap. So, the tensile strain induced blueshift in optical absorption spectra or infrared extinction spectra of BP [144].

**Fig. 8:**
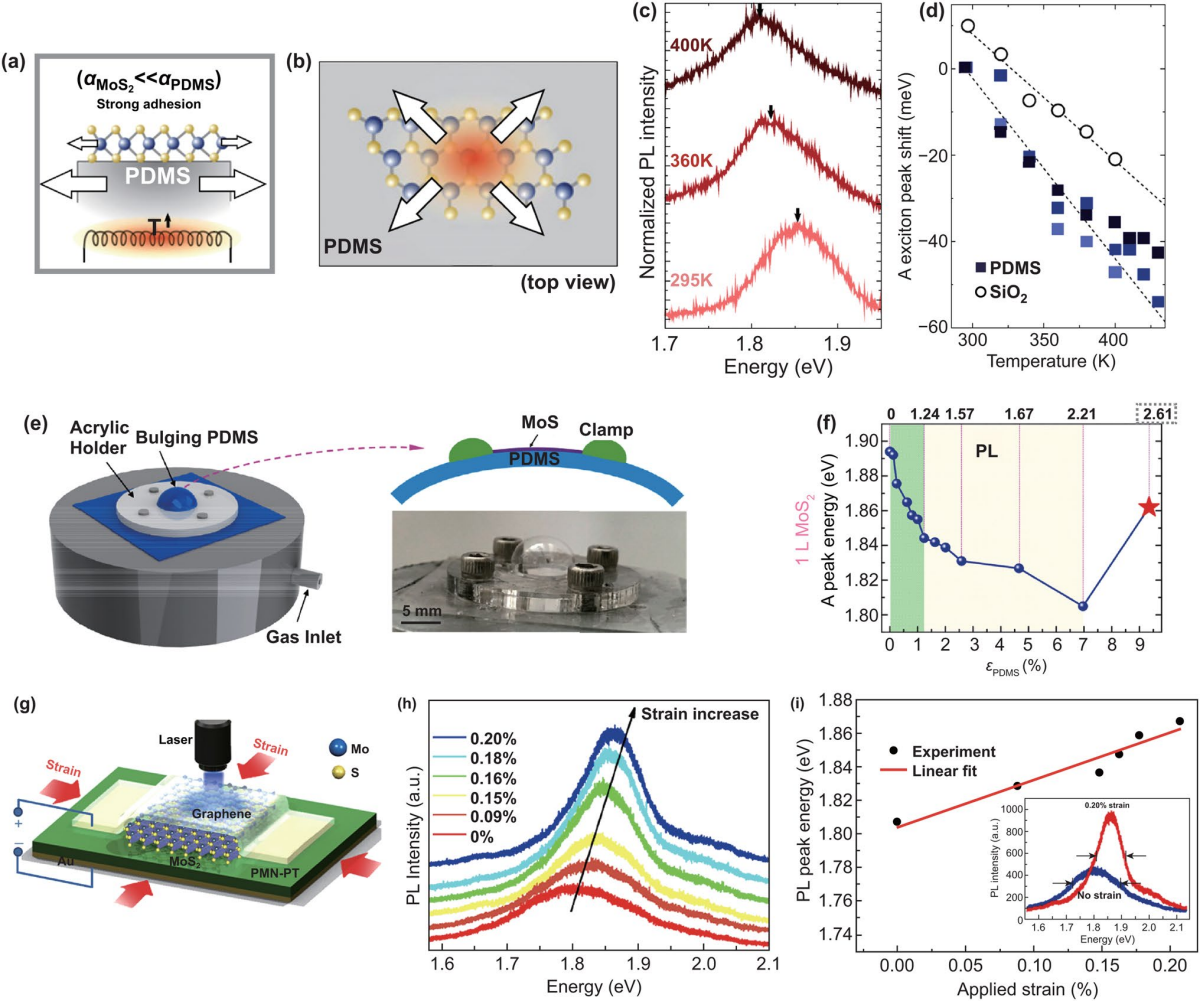
Applied biaxial strain to 2D semiconductors. **a** Schematic of applied biaxial strain on MoS_2_ through heating PDMS substrate. **b** Top view of a monolayer MoS_2_ on PDMS substrate. **c** Temperature-dependent PL spectra of MoS_2_ on PDMS substrate. **d** Temperature-dependent shift of the A exciton peak position on PDMS and SiO_2_ substrates. Reproduced with permission [145]. Copyright 2015, IOP Publishing. **e** Measurement setup of blown-bubble bulge technique. **f** Extracted PL peak position shift with applied strain in PDMS. Reproduced with permission [146]. Copyright 2017, American Chemical Society. **g** Schematic of applied biaxial compressive strain to 2D MoS_2_. Effect of biaxial compressive strain on the PL spectra **h** and PL peak energy **i** of the MoS_2_. Reproduced with permission [148]. Copyright 2013, American Chemical Society

Uniaxial strain is usually and easily applied to the 2D material, there are also some ingenious experimental designs can apply biaxial strain to the 2D material. In 2015, Plechinger et al. [145] transferred the monolayer MoS_2_ on the large thermal expansion PDMS substrate and applied biaxial tensile strain on the sample through heating the substrate; Fig. 8a, b shows the experiment schematic. The PL (Fig. 8c) was used to estimate the strain, larger redshift of MoS_2_ on PDMS as compared to SiO_2_ substrates (Fig. 8d). They also found that 0.2% biaxial tensile strain could be introduced by SiO_2_ substrate, which will result in a band gap change of 105 meV/% biaxial tensile strain. Yang et al. [146] transferred 2D MoS_2_ on PDMS substrate and applied the biaxial tensile strain to the sample supported by a novel blown-bubble bulge technique, the measurement setup as shown in Fig. 8e. The strain is isotropic biaxial at < 1.2% strain and becomes anisotropic because of the sliding at larger strains. PL spectroscopy of monolayer MoS_2_ shows that the band gap decreases with increasing strain, and the fracturing strain is about ~ 9.4%, as shown in Fig. 8f. The similar work is also reported by Lloyd et al. [147]. And Hui et al. [148] applied biaxial compressive strain to a 3L MoS_2_ by transfer it on a piezoelectric substrate, as shown in Fig. 8g. The largest applied compressive strain is about 0.2%. The PL results in Fig. 8h, i show that the blueshift of the direct band gap for ∼ 300 meV/% strain and the PL intensity is also enhanced.

### Applying Strain by Creating Wrinkles in 2D Semiconductors

**Fig. 9:**
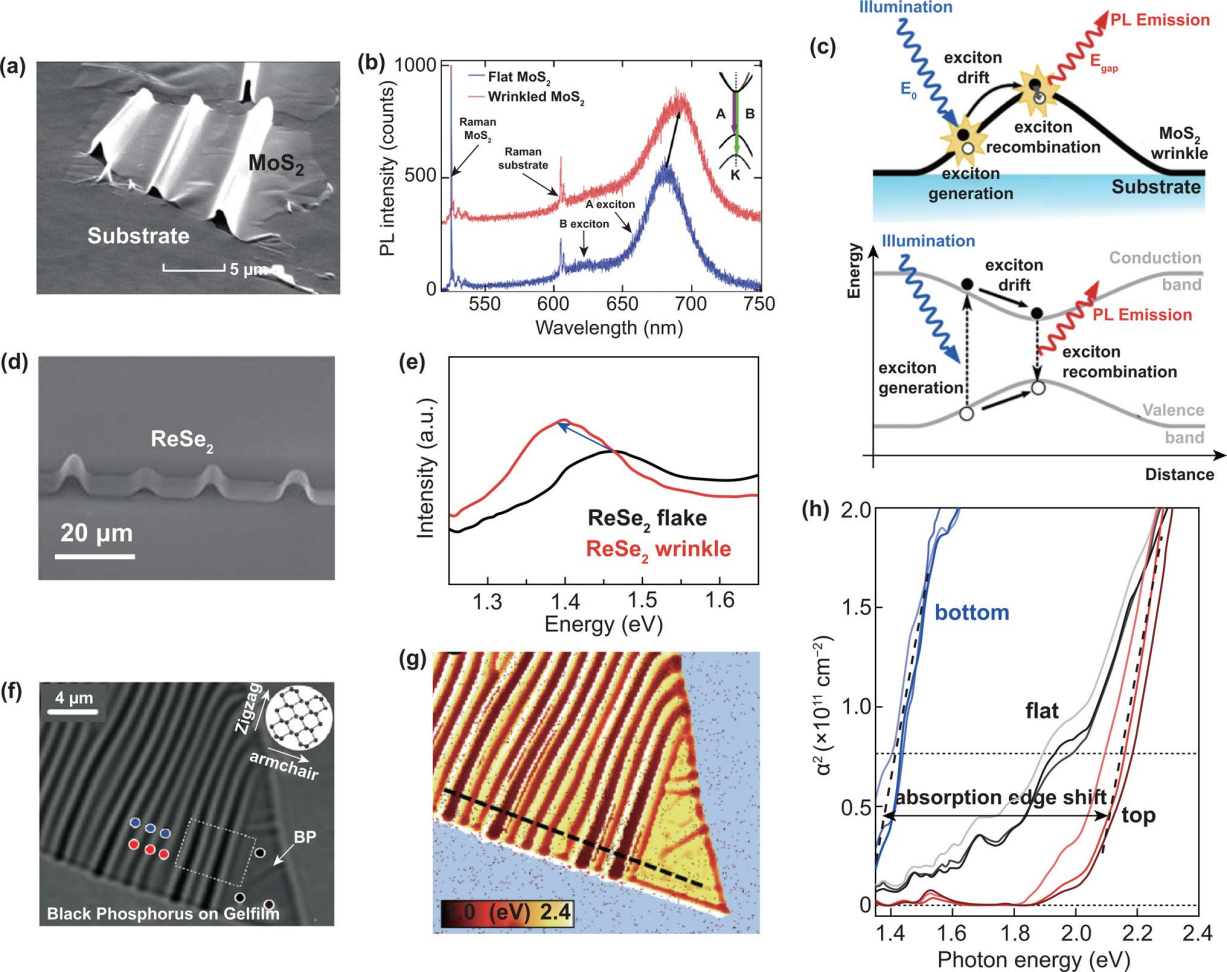
Observed piezoresistive effect in wrinkles 2D materials. The SEM image of **a** MoS_2_ and **d** ReSe_2_ wrinkles. PL spectra measured on the flat and on the wrinkle regions of **b** MoS_2_ and **e** ReSe_2_. **c** Band diagram in the wrinkled MoS_2_. **a–c** Reproduced with permission [152]. Copyright 2013, American Chemical Society. **d, e** Reproduced with permission [153]. Copyright 2015, American Chemical Society. **f** Optical image of a wrinkled 10-nm-thick black phosphorus flake and **g** corresponding optical absorption mapping. **h** Optical absorption spectra acquired on ripple summits, valleys and flat regions. Reproduced with permission [154]. Copyright 2016, American Chemical Society

Large localized uniaxial strain exists in the wrinkles 2D semiconductors. Transferring the sample to an elastomeric substrate and stretched, the wrinkles would be formed during the tension releasing process thanks to the mismatch of elastic modulus between 2D materials and elastomeric substrate. This method has been used for applied strain on graphene and nanoribbons early [149–151]. Figure 9a shows the SEM image of wrinkles of 3–5 layers MoS_2_, and the largest uniaxial strain in the wrinkles part was estimated at 2.5%. A redshift of PL spectra was observed in wrinkles part of MoS_2_, as shown in Fig. 9b, which suggests that the strain reduced the band gap (~ 90 mV/%). The band structure as shown in Fig. 9c reveals that photogenerated excitons move to the area when applying higher strain. The simulation results based on a tight-binding theory were consistent with the experimental observations [152]. The method was also carried out on a 2D ReSe_2_, a sample with wrinkles as shown in Fig. 9d. Except for the redshift of PL spectra (Fig. 9e), the electrical properties were also be changed and induced magnetism [153]. Quereda et al. [154] used the same method to modulate the optoelectronic properties multilayer BP, and the optical image and corresponding optical absorption mapping of a wrinkled 10-nm BP are displayed in Fig. 9f, g, respectively. The band gap in Fig. 9h extracted from the optical absorption spectra shows a ∼ 0.7 eV shift between the +10% tensile strain region and − 30% compressive region, which is greatly larger than strain adjustable range reported for 2D TMDs. Besides, these experimental results are good agreement to theoretical models.

Recently, Du et al. [155] used the same method and found that the 2D PbI_2_ multilayer maintains a direct band gap nature under a large experimental strain up to 7.69%. The strained 2D PbI_2_ offers reference for potential optoelectronic device applications. The wrinkles also introduced in massive reduced graphene oxide [156], WS_2_ [157], and Bi_2_Se_3_/Bi_2_Te_3_ heterostructure [158] by the similar method; as well, the theoretical foundation is getting better and better [159, 160].

**Fig. 10:**
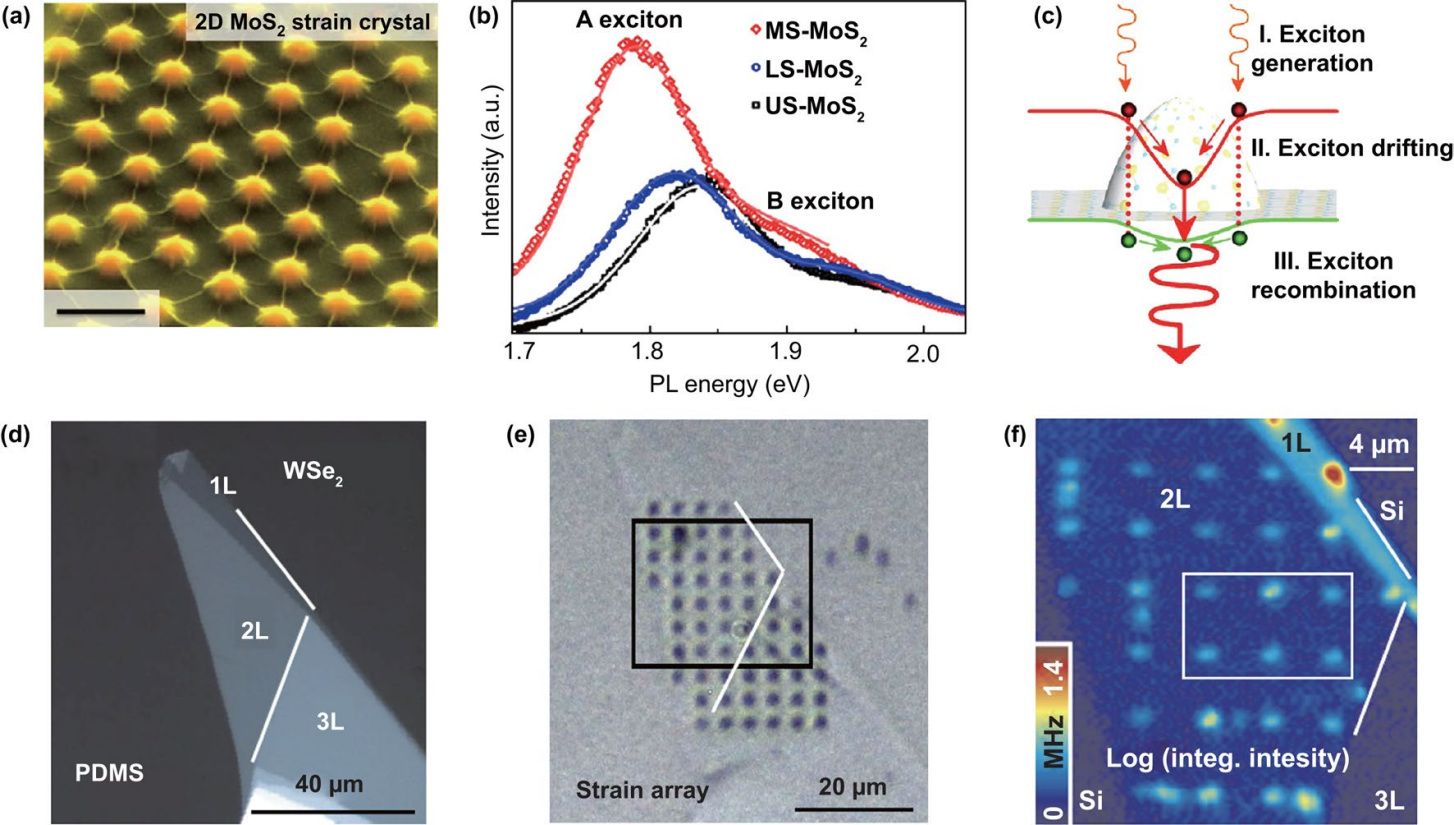
Applied biaxial strain by transfer 2D materials to patterned substrates. **a** SEM image of the 2D MoS_2_ transferred onto a nanocone array substrate; scale bar is 100 nm. **b** PL spectra of most strained, less strained and unstrained 2D MoS_2_. **c** Schematic of the funnel effect in strained MoS_2_. Reproduced with permission [162]. Copyright 2015, Nature Publishing Group. Optical micrograph of 2L WSe_2_ **d** before and **e** after the transfer onto the nanopillars substrate. **f** PL mapping of the WSe_2_ on nanopillars substrate. Reproduced with permission [163]. Copyright 2017, Nature Publishing Group

The fixed and repeatable local strain can be applied to 2D materials by transferring them onto patterned substrates [161]. For example, Li et al. [162] transferred the monolayer MoS_2_ on a patterned nanocone array substrate to applied biaxial tensile strain; the SEM image of sample is shown in Fig. 10a. The monolayer MoS_2_ on top of the nanocones is applied high strain, and the strain gradually decreases from the highest part to the flat part. Figure 10b shows the strong arise and redshift (~ 50 meV) of PL peak at top region of monolayer MoS_2_ (~ 1% biaxial tensile strain). The increased peak intensity of PL spectra is due to the exciton funnel effect. A built-in electric field pointing from the top to the bottom of the nanocone is produced due to the difference in band gap at top and bottom, as illustrated in Fig. 10c. In consequence, photogenerated excitons are driven to the top region of the nanocones, leading to the enhancement of PL peak. The efficient funneling of excitons in mono- and bilayer WSe_2_ was also observed by using the similar method [163]. The mechanically exfoliated WSe_2_ was transferred from a PDMS substrate (Fig. 10d) to a substrate with nanopillars (Fig. 10e). The PL mapping (Fig. 10f) shows the higher peak intensity at the top of nanopillars due to the exciton funnel effect. Recently, Sortino et al. [164] also introduced strain in 2D WSe_2_ by transferring them on a substrate with dielectric nanoantennas. The redshift of PL spectra was observed for decreasing the size of nanoantennas, indicating the increase in strain in WSe_2_. The largest achieved strain is up to 1.4% for monolayer WSe_2_ and 3% strain for bilayer WSe_2_. This method has the prospect of manufacturing integrated circuits based on strained 2D materials. And the strain is increased as the radius decreases based on this method, and micro–nanoprocessing technology may limit the use of this method. And growing the 2D crystals on the surface of nanospheres or quantum dots may achieve large strained sample.

When the relationship between PL and strain is clarified, the PL can be used as a tool to probe the magnitudes of strain in 2D semiconductor. Based on the study of the effect of strain on the PL spectrums of 2D materials, it was found that the growth substrates can also introduce strain to 2D materials. Chae et al. [165] reported that the substrate-introduced built-in strain in the CVD-grown MoS_2_ can be quantitatively probed by PL spectroscopy. The 0.4% biaxial tensile strain was observed in the monolayer MoS_2_ on SiO_2_ substrate, while only ~ 0.2% strain was observed in the sample on the sapphire, mica and h-BN substrate. The origin of tensile strain in CVD-grown 2D layers is not only due to the thermal expansion coefficient mismatch between the layer and the substrate, but also due to deviation from the weak vdW interaction at the interface that prevents slippage. Ahn et al. [166] use the similar method to investigate the induced build-in strain in monolayer WSe_2_ grown on different substrates. The shift of PL peaks showed 1% biaxial tensile and 0.2% biaxial compressive strains in monolayer WSe_2_ grown on SiO_2_ and strontium titanate substrate, respectively.

Based on similar principles, depositing additional layers is another way to introduce built-in strain in 2D materials. A vertically stacked carbon–MoS_2_ nanosheet was synthesized by Oakes et al. and an average 0.1% compressive strain propagates into the MoS_2_ nanosheet lattice due to lattice mismatch in the carbon and MoS_2_ interface [167]. Hazarika et al. [168] used colloidal atomic layer deposition grown core–shell CdS/CdSe nanoplatelet heterostructures with atomic precision. The strain in core and shell can be modulated by changing the number of layers of core or shell. The optical gap redshifted by 446 meV was observed in core CdSe when the number of layers of shell increases from 0 to 4, which indicates that large strain is obtained and the strain can be controlled by changing the thickness of the shell material. The strain in shell layer was not discussed which is also worth studying.

A summary of strain tuning the optical band gap of 2D semiconductors is shown in Table 1. So far, the experimentally applied strain can reach to 5% and the changing coefficient of direct band gap is less than indirect band gap whose coefficient value is marked with (I) in the table. Most of the experiments were carried out on 2D TMDs, while the larger changing coefficient of PL spectrum in 2D BP and InSe was observed recently, indicating their application potential in strained electronics. Ingenious method of applying compressive stress can increase the intensity of PL, suggesting their application potential in strain optic-electronics. Interestingly, the MoS_2_ and WS_2_ in a MoS_2_/WS_2_ van der Waals heterostructure show approximate PL changing coefficient under strain, which is more than three times in difference when tested separately. This provides an idea to employ strain modulate the optoelectronic performance of 2D van der Waals heterostructures by piezoresistive effect.

**Table 1:** Comparison between the different strain experimental results available in the literature for 2D semiconductors

Materials	Thickness	Strain	Peak shift	Coefficient (meV/%)	Refs.
MoS_2_	1 L	2.2% uniaxial tensile	Redshift	45	[40]
MoS_2_	2 L	2.2% uniaxial tensile	Redshift	120	[40]
MoS_2_	1 L	Tensile	Redshift	77.3 ± 10	[171]
MoS_2_	2 L	Tensile	Redshift	116.7 ± 10	[171]
MoS_2_	3 L	Tensile	Redshift	22.7 ± 6	[171]
MoS_2_	3 L	0.2% uniaxial tensile	Redshift	300 (I)	[127]
MoS_2_	1 L	0.52% uniaxial tensile	Redshift	64 ± 5	[134]
MoS_2_	2 L	0.52% uniaxial tensile	Redshift	71 ± 5 77 ± 5 (I)	[134]
MoS_2_	1 L	0.8% uniaxial tensile	Redshift	48	[135]
MoS_2_	2 L	0.8% uniaxial tensile	Redshift	46 86 (I)	[135]
MoS_2_	1 L	0.2% biaxial tensile	Redshift	105	[145]
MoS_2_	1 L	1.2% biaxial tensile	Redshift	18.5	[146]
MoS_2_	3 L	1.2% biaxial tensile	Redshift	27.3 89.1 (I)	[146]
MoS_2_	3L	0.2% biaxial compressive	Blueshift	300	[148]
MoS_2_	3–5L	2.5% uniaxial tensile	Redshift	60	[152]
MoS_2_	1 L	1% biaxial tensile	Redshift	50	[162]
MoS_2_	1 L	5% biaxial tensile	Redshift	99	[147]
MoS_2_	2 L	5% biaxial tensile	Redshift	91 144 (I)	[147]
MoS_2_	3 L	5% biaxial tensile	Redshift	73 110 (I)	[147]
MoS_2_	1 L	0.48% biaxial tensile	Redshift	135	[213]
WSe_2_	1 L	1.4% uniaxial tensile	Redshift	54	[139]
WSe_2_	2–4 L	2% uniaxial tensile	Redshift	60–70	[140]
WSe_2_	Multilayer	1.35% uniaxial tensile	Redshift	50–75	[225]
ReSe_2_	1 L	1.64% uniaxial tensile	Redshift	70	[153]
WS_2_	1 L	2.2% uniaxial tensile	Redshift	11 19 (I)	[137]
MoSe_2_	1 L	1.1% uniaxial tensile	Redshift	27	[138]
BP	6 L	0.92% uniaxial tensile	Blueshift	117–124	[141]
BP	10 nm	5% uniaxial tensile	Blueshift	140	[154]
BP	20 nm	0.8% uniaxial tensile	Blueshift	99–109	[132]
BP	3L	0.3% biaxial tensile	Blueshift	158	[144]
BP	4L	0.3% biaxial tensile	Blueshift	185	[144]
InSe	4 nm	1.06% uniaxial tensile	Redshift	153	[142]
InSe	5 nm	0.62% biaxial compressive	Blueshift	140	[142]
PbI_2_	100 nm	7.69% uniaxial tensile	Redshift	5.8	[155]
MoS_2_/WS_2_	1L/1L	0.7% uniaxial tensile	Redshift	68 (MoS_2_) 63 (WS_2_)	[226]
MoS_2_/WS_2_	1L/1L	0.7% uniaxial biaxial compressive	Blueshift	36 (MoS_2_) 24 (WS_2_)	[226]

### Study Strain-Engineered 2D Semiconductors by AFM Apparatus

#### Applying Strain by AFM Tip

**Fig. 11:**
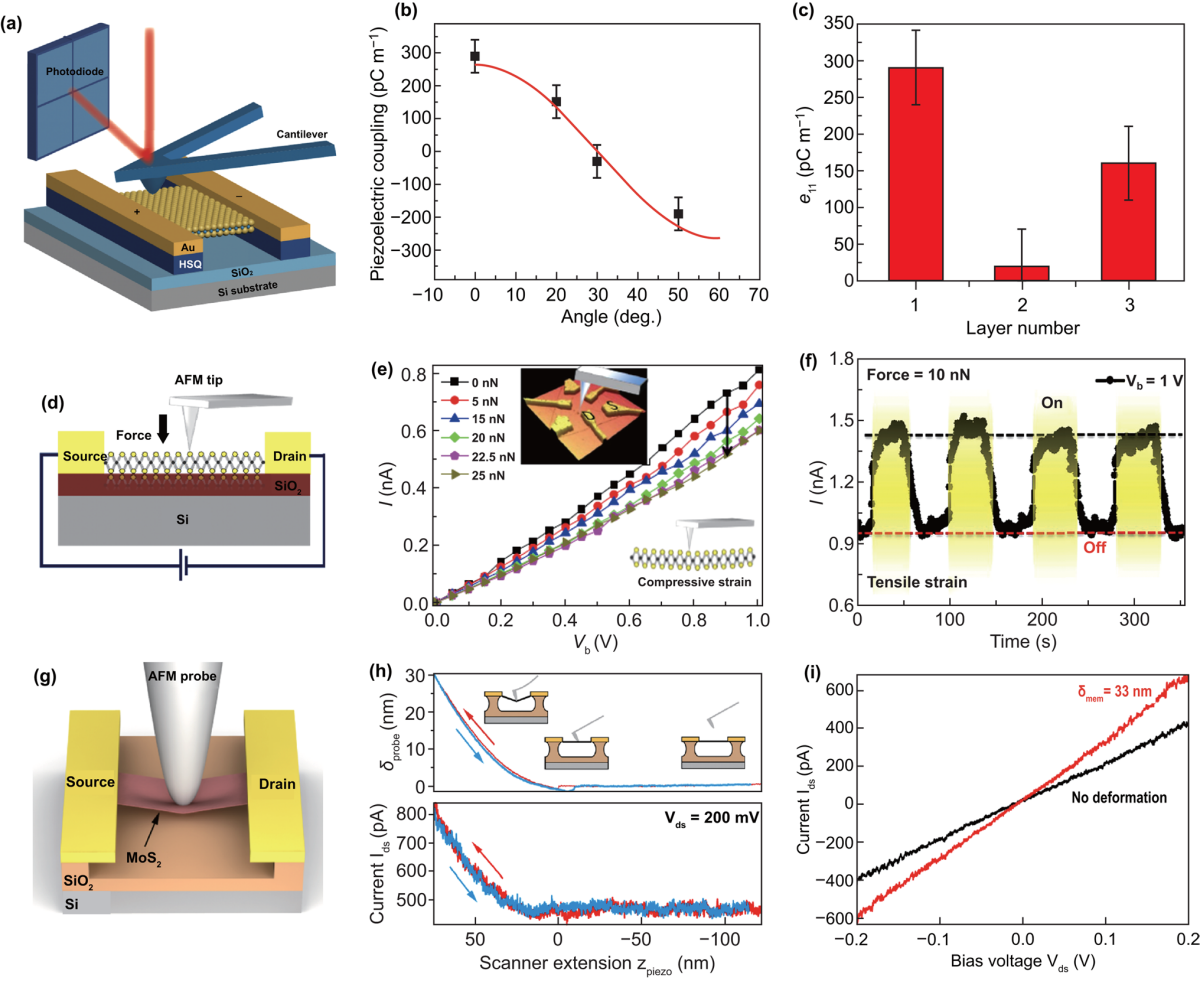
Applied strain to 2D semiconductors by AFM tip. **a** Schematic drawing of measuring piezoelectric property of monolayer MoS_2_ by using an AFM probe. **b** Measured piezoelectric coupling strength as a function of the crystal’s azimuthal angle *θ*. **c** Measured piezoelectric coefficient in one-, two- and three-layer MoS_2_. Reproduced with permission [169]. Copyright 2014, Nature Publishing Group. **d** Schematic illustration of the measurement setup. **e** *I*–*V* characteristics of the MoS_2_ device at different applied forces. **f** Current response of CVD monolayer MoS_2_ device at repeated force at a fixed bias voltage of 1 V. Reproduced with permission [170]. Copyright 2015, Nature Publishing Group. **g** Schematic drawing of the experiment setup for electrical performance characterization of a suspended MoS_2_ devices under the strain applied by AFM tip. **h** Cantilever deflection and the drain current of the device as a function of the piezo-scanner extension. **i** *I*–*V* curve of the device under the strain by AFM tip and without deformation. Reproduced with permission [171]. Copyright 2017, American Chemical Society

AFM apparatus can be used for quantitatively applying force to the nanomaterials by the probe. Almost at the same time as the work of Zhong Lin Wang group was published, Xiang Zhang group [169] also observed the piezoelectric effect in free-standing monolayer MoS_2_ by using an AFM. Figure 11a shows the measurement setup. The anisotropy of piezoelectricity in monolayer MoS_2_ was also observed. Since the crystal symmetry belongs to the *D*_3h_ group, the piezoelectric coefficients of the monolayer MoS_2_ are functions of crystal’s angle with a period of 120°, as shown in Fig. 11b. Figure 11c shows the thickness dependence of piezoelectric coefficient, which confirms the piezoelectricity only exists in odd number layers MoS_2_. The measured piezoelectric coefficient of *e*_11_ is up to 2.9 × 10^−10^ C m^−1^.

The monolayer MoS_2_ synthesized by the CVD method is a regular triangle with three zigzag edge, which makes it easier to confirm the crystal orientation with piezoelectric effect. We observed the piezoelectric phenomenon in a CVD-grown triangular monolayer MoS_2_ by using AFM for the first time, Fig. 11d shows the measurement setup [170]. The results show that the source-drain current of the MoS_2_ devices can be regulated by strain variation (Fig. 11e). The *I*–*t* curves in Fig. 11f show the good current response of the device to the repeated force. A built-in electric field pointing from the edge to center is created in the strain sample due to the piezoelectric effect and produce polarization carriers which lead to the varies of SBH of MS contacts, resulting in the decrease in SBH with the increase in tensile strain and the increase in SBH with the increase in compressive strain.

The piezoresistive effect of 2D MoS_2_ was also observed by using an AFM by Manzeli et al. [171]; the experiment setup is shown in Fig. 11g. The deflection of AFM tip is controlled by a piezoelectric device, and the drain current is unchanged when the tip gradually approaches to the sample and begins to increase when the tip has touched the sample (Fig. 11h). The drain current is increased and the resistance is decreased under the deformation (Fig. 11i). The results show that the piezoresistive GF of monolayer, bilayer and trilayer MoS_2_ is about − 148, − 224, and − 43.5, respectively.

#### Study Piezoelectric Effect by PFM Mode

**Fig. 12:**
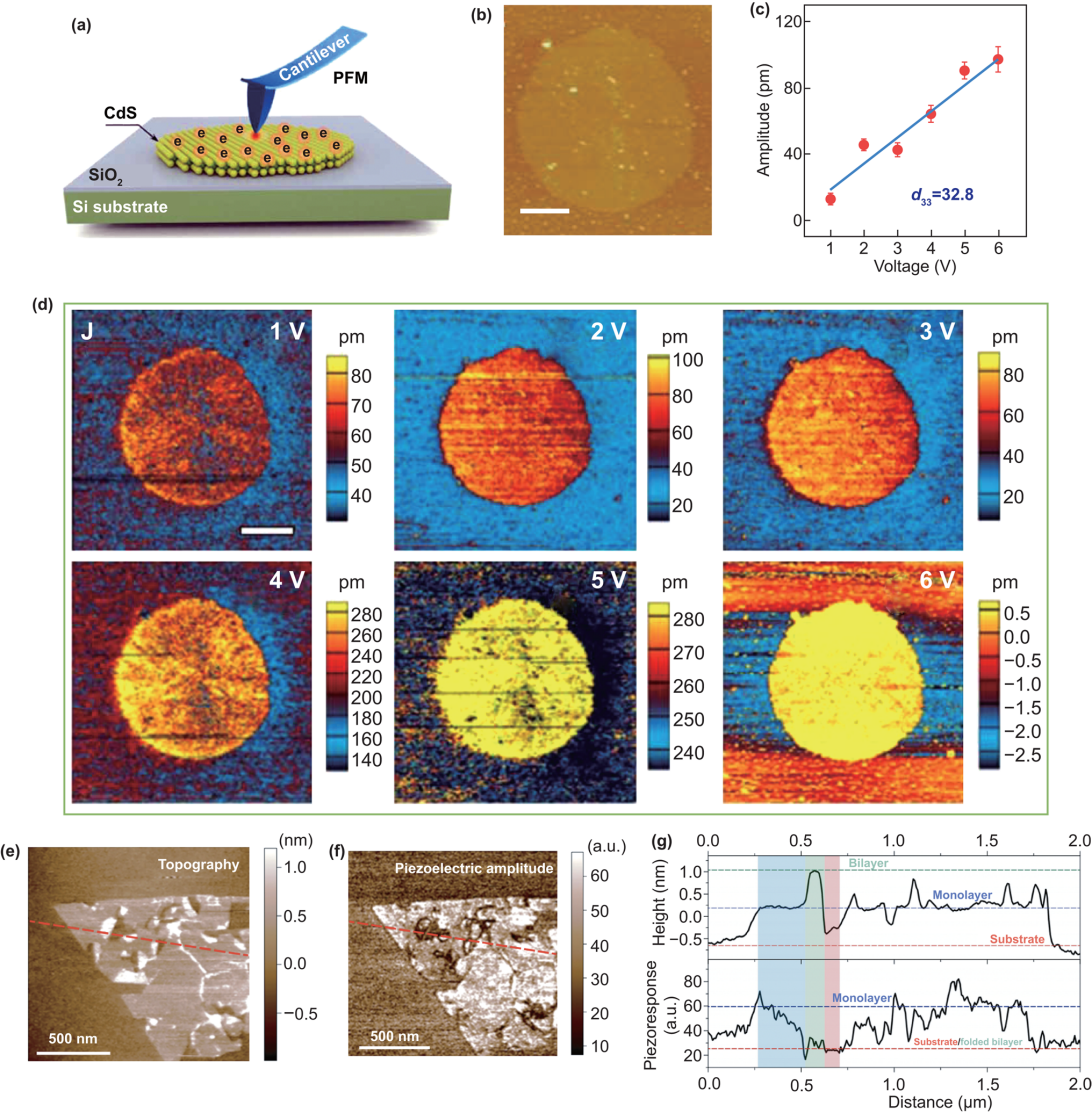
Observing out-of-plane piezoelectric effect by PFM mode. **a** Schematic illustration of PFM measurement. **b** AFM topography images of the 2D CdS. **c** Average amplitude variations versus applied voltages. **d** Piezoelectric amplitude images observed by PFM technology with tip voltages from 1 to 6 V. Reproduced with permission [173]. Copyright 2018, AAAS. **e** Topography and **f** piezoelectric amplitude image a monolayer Janus MoSSe. **g** The step height and piezoelectric amplitude profile along the dashed lines in **e, f**. Reproduced with permission [94]. Copyright 2017, Nature Publishing Group

According what we have learned above, the in-plane strain was applied on a two-terminal device by an AFM tip, which can only be used to study in-plane piezoelectric coefficients, such as *d*_11_ and *d*_22_. Vertical piezoelectric coefficients, such as *d*_33_ and *d*_31_, are also important to some 2D piezoelectric materials. Piezoelectric force microscopy (PFM) is one of the AFM modes, which can be used to investigate the vertical piezoelectric properties of nanomaterials [172]. In 2016, the vertical piezoelectricity of 2D CdS was evaluated by using a PFM [173]. Figure 12a shows the measurement setup. The topography images of a 3-nm-thickness CdS and corresponding piezoelectric potential mapping with various applied voltages are shown in Fig. 12b, d. The extracted data in Fig. 12c suggest that the piezoelectric coefficient d_33_ of 2D CdS is up to 32.8 pm V^−1^, which is four-time performance of the bulk CdS. In 2017, Lu et al. [94] synthesized the monolayer Janus MoSSe by CVD method and they use PFM to observe the piezoelectric effect in vertical of the monolayer sample and the piezoelectric coefficient d_31_ is ∼ 0.1 pm V^−1^. Figure 12e, f displays the topography and piezoelectric amplitude mapping image of a MoSSe sample with monolayer and bilayer region. The step height and piezoelectric amplitude profile in Fig. 12g shows that the piezoelectric effect for monolayer MoSSe is larger than that of bilayer region. The out-of-plane piezoelectricity of 2D α‑In_2_Se_3_ [174] and CuInP_2_S_6_ [175] was also be investigated by PFM. The piezoelectric response was also observed in a two-terminal monolayer MoS_2_ [176] and odd layer WSe_2_ [177] device by PFM, and the amplitude produced in out-of-plane is originated from the Poisson’s effect in 2D materials.

#### Study Strain-Engineered Vertical Transport Behavior by C-AFM Mode

**Fig. 13:**
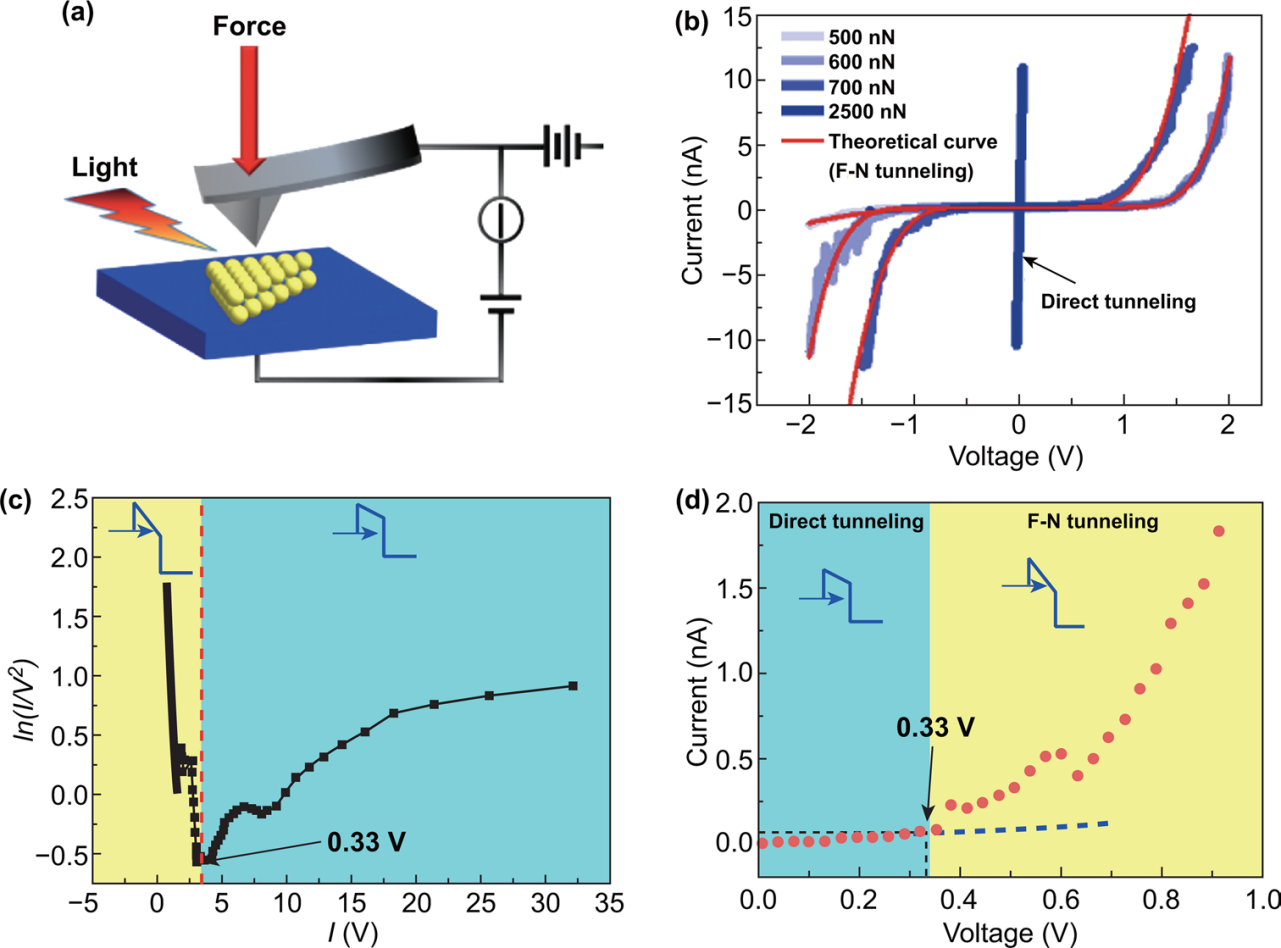
Strain tuning vertical transport behavior by C-AFM mode. **a** Schematic of the measurement setup used to obtain the vertical electrical transport property curves of MoS_2_. **b** *I*–*V* curves measured under different force. **c** ln (*I*/*V*^2^) versus 1/*V* curves and **d** local *I*–*V* curves in the forward bias for distinguishing between direct tunneling and F-N tunneling models. Reproduced with permission [180]. Copyright 2018, IOP Publishing

The conductive AFM (C-AFM) is also one of the AFM modes, and it can be used to measure the current when applied force on the conductive probe. Yang et al. [178] observed tunneling phenomena in 2D materials by using a C-AFM. Fu et al. [179] used a C-AFM apparatus, taking conductive probe and the conductive substrate as source and drain electrodes, and found that the vertical resistance can be modulated to attain four orders of magnitude by applying various tip forces thanks to the increase in the tunneling current when the tunneling layer (MoS_2_) is compressed under the tip force. More systematic work was published by our group [180] using the similar method, and Fig. 13a shows the schematic of the measurement setup. The Simmons approximation was used to fit the measured *I*–*V* curves (Fig. 13b), which reveals that the transport behavior is direct tunneling at low bias and Fowler–Nordheim tunneling at high bias, and the transition voltage is extracted (Fig. 13c, d). The effect of light on the tunneling current of vertical MoS_2_ is also investigated. We believed that the light can modulate the tunneling current by photothermal effect and photoelectric effect. Jorge et al. also reported a similar work, while they believe that the transport behavior of tip/MoS_2_/ITO junction is a double Schottky barrier model and rectifying behavior can be tuned by tip force [181]. In order to clarify the mechanism and characteristics of the force, electric and photon coupling, the vertical transport mechanism still needs further study and the C-AFM is a good experiment method.

### Apply Strain by Surface Acoustic Wave

The separation of electron and holes caused by the piezoelectric effect will change the in-plane carrier distribution of the 2D materials. The electrons will move to one side and the holes will move to another side drive by piezoelectric potential, leading to more exciton emission in one side and more trion emission in another side. Therefore, the PL spectrum is likely to observe piezoelectric phenomena, but rarely reported.

**Fig. 14:**
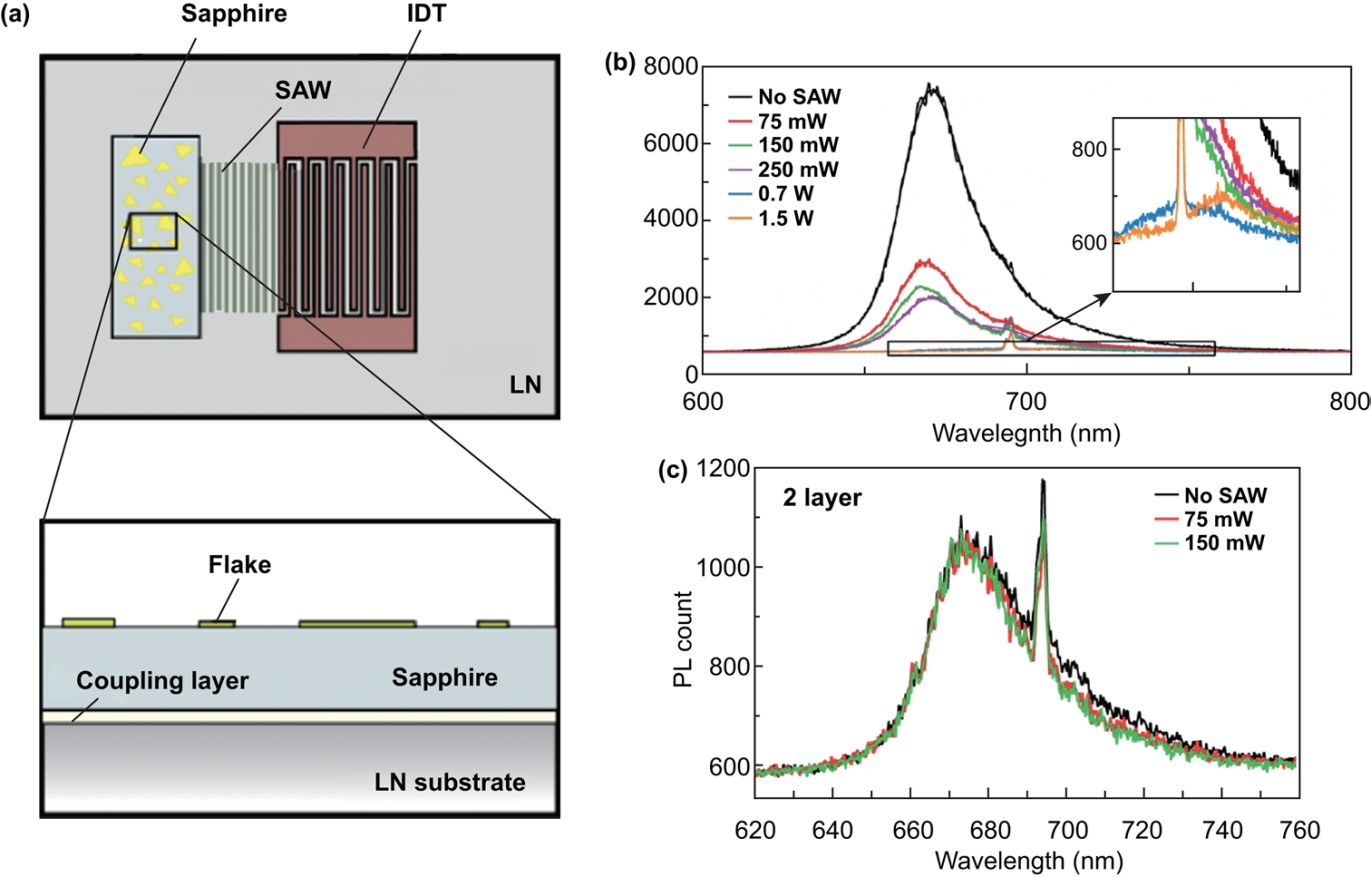
Applied strain to 2D semiconductors by surface acoustic wave. **a** Schematic illustration of the experimental setup comprising the SAW device, which consists of IDT electrodes photo-lithographically patterned on a lithium niobate substrate. PL spectra of **b** a monolayer and **c** bilayer MoS_2_ flake with respect to a range of SAW input powers. Reproduced with permission [182]. Copyright 2017, American Chemical Society

A novel experiment is reported by Rezk et al. [182], and they use a surface acoustic wave (SAW) device (Fig. 14a) to induce a propagating space periodic strain in 2D MoS_2_ and measured PL spectrum simultaneous. Figure 14b shows a strong quenching in the PL which was clearly observed in odd number layer MoS_2_ on 75 mW SAW excitation. And the relative ratio of the trion to exciton peak intensities reduces with increasing SAW power. No quenching in the PL was observed in even number layers of MoS_2_, and PL results for bilayer MoS_2_ are shown in Fig. 14c. A acoustically electric field will be generated due to the inherent piezoelectric properties of odd layer MoS_2_, and the PL quenching is significantly weaker for three-layered MoS_2_ because of a weaker piezoelectric electromechanical coupling coefficient compared to its single-layer counterpart. This article shows that the PL spectra can also be used to investigate the piezoelectric phenomena in 2D semiconductors.

Compared with bulk and one-dimensional materials, 2D materials have a simpler crystal structure, making it easier to theoretically estimate their properties. The piezoelectric coefficients of monolayer MoS_2_ is predicted to be 3.73 pm V^−1^ (*d*_11_) and 3.64 × 10^−10^ C m^−1^ (*e*_11_) by first-principle calculations [73]. And the experimental results show that the d_11_ and e_11_ of monolayer MoS_2_ is 3.78 pm V^−1^ [176] and 2.9 × 10^−10^ C m^−1^ [169], which are very close to the theoretical results, while the *d*_31_ of MoSSe is about 0.1 pm V^−1^ observed by PFM [94], which is larger than the theoretical prediction of 0.02 pm V^−1^ [95]. The theoretical prediction of band gap shift and indirect–direct band gap transition were also proved experimentally. However, the change in rate of the band gap with strain was observed in a large range in different articles, whose value is between 48 and 105 meV/% strain for monolayer MoS_2_, as shown in Table 1. This may be due to the difference in crystal quality. Overall, the experimental observations are consistent with the theoretical predictions, and the simulation studies help to deepen understanding of theories in strained 2D semiconductors.

In this section, we have summarized the experimental research progress of strain-engineered 2D materials from the perspective of the methods of applied strain. The main methods of applying strain to 2D materials include bending flexible substrate, creating wrinkles and using AFM apparatus. The various spectrum, electrical and piezoelectric amplitude images can be used to analyze the piezoelectric effect and piezoresistive effect. Although a variety of methods for applying strain on 2D materials have been developed, most of them are limited to experimental observation and verification, while not suitable for the applications in electronic components. The new techniques, which can permanently and easily integrate applied strain on the devices based on 2D semiconductors, need to be developed. With the expansion of the 2D materials, such as wurtzite structure, Janus 2D materials and monolayer SnSe and SbAs which have been predicted to process highest piezoelectric coefficient, their piezoelectric properties urgently need to be observed experimentally.

## Applications of Strain-Engineered 2D Semiconductors

The 2D materials are famous for their special electricity, photonics, mechanics and thermal properties. These 2D semiconductors have been widely used for ultrafast pulse laser generation [183–186], optical switching [26, 187, 188], optoelectronics [189, 190], biophotonics [32, 191, 192], and energy [193–195]. Therefore, it is necessary to master the physical and chemical properties about 2D materials. In this section, we will highlight their application of stain-induced piezoelectric effect and piezoresistive effect in strain sensors, photodetectors and nanogenerators.

### Strain Sensors

Flexible and wearable strain sensor is becoming more and more important with the development of Internet of things, which shows widespread applications in health monitoring, e-skin, intelligent electronics, etc. One of the most important applications of piezoelectric and piezoresistive effects in 2D materials is the strain sensor, which can meet the requirements of future applications.

In 2015, Manzeli et al. [171] presented the high GF data of 2D MoS_2_. Subsequently, strain sensors take advantage of the piezoresistive effect of 2D semiconductors were successively developed. In 2016, Park et al. [196] reported a large-area (2.2 × 2.2 cm^2^), conformal tactile sensor based on CVD-grown bilayer MoS_2_ continuous film. The piezoresistive MoS_2_ strain sensor exhibited good optical transparency (over 80%), mechanical flexibility (1.98%) and high GF (72.5). Recent results show that the GF of mechanical exfoliation few-layer MoS_2_ [197] and large-area monolayer MoS_2_ continuous film [198] is up to 240 and 104, respectively.

**Fig. 15:**
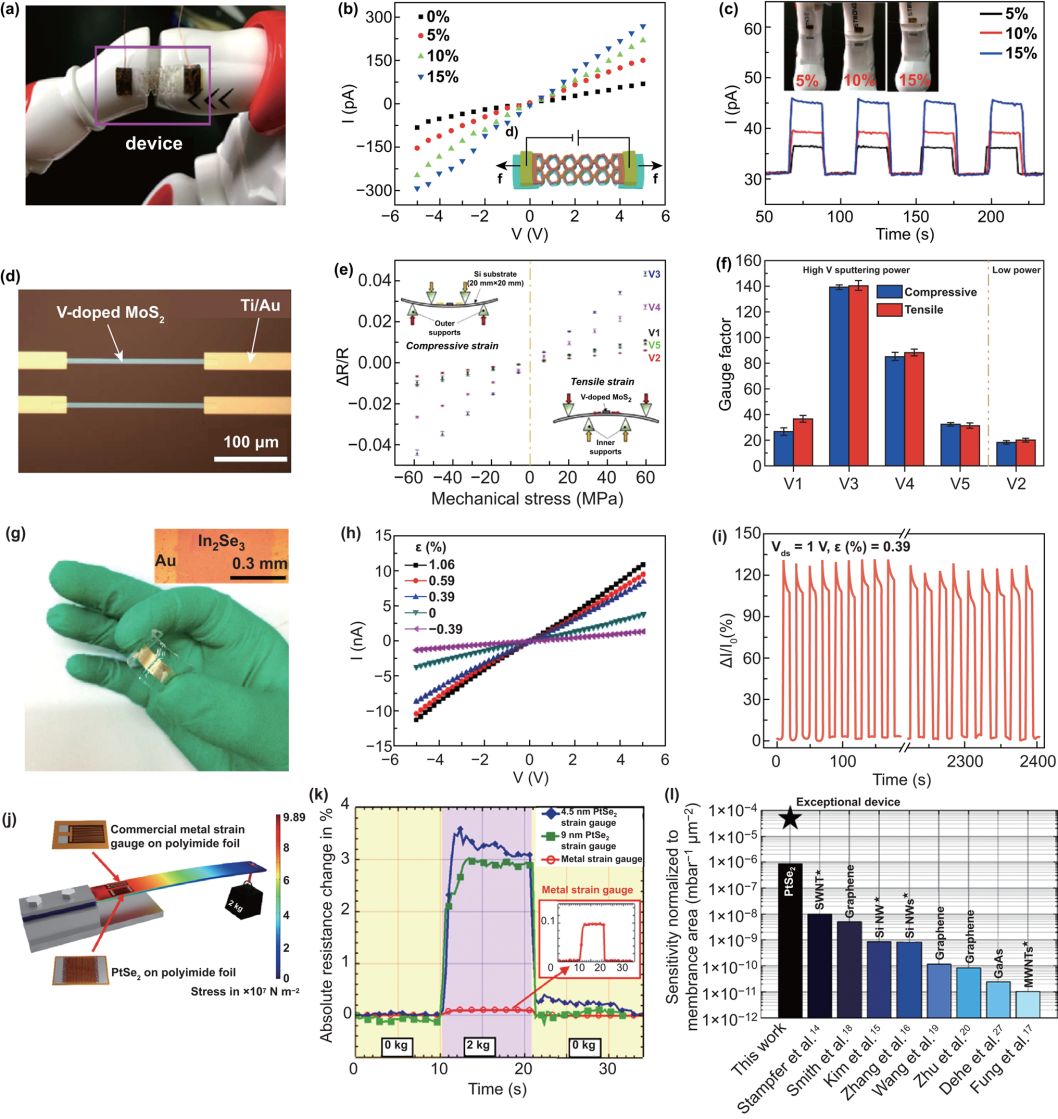
Piezoresistive strain sensor based on 2D semiconductors. **a** Kirigami structure devices on the surface of elbow of a robot. **b** *I–V* curves of the devices under different deformation rates. **c** Strain sensor based on the Kirigami structure devices and the time-dependent response of devices. Reproduced with permission [199]. Copyright 2018, American Chemical Society. **d** Optical image of the V-doped MoS_2_ strain sensor. **e** Change in resistance as function of strain. **f** Relationship between V-doping concentration and GFs of the sensor. Reproduced with permission [198]. Copyright 2019, The Japan Society of Applied Physics. **g** Optical image of a 2D In_2_Se_3_ strain sensor. **h** Device currents under both uniaxial tensile and compressive strain. **i** Multicycle normalized current change in the sensor under uniaxial tensile strain. Reproduced with permission [200]. Copyright 2016, American Chemical Society. **j** Bending beam setup with applied 2D PtSe_2_. **k** Electrical readout signal during the measurement with an absolute resistance change against time showing two PtSe_2_ devices. **l** Comparison of the sensitivity with other pressure sensors. Reproduced with permission [201]. Copyright 2018, American Chemical Society

MoS_2_ strain sensor is well developed due to the advances in CVD preparation technology. However, the GF of CVD MoS_2_ sensor is low and can only withstand a limited mechanical strain (< 2%). Zheng et al. [199] developed a kind of strain sensor based on MoS_2_ Kirigami structures which was made by using a plasma etching approach. The strain sensor can be used for the elbow and knee joint of a robot, as shown in Fig. 15a. The MoS_2_-based Kirigami strain sensor can withstand 15% strain (Fig. 15b), which is only 0.75% for the sensor based on as grown sample. Figure 15c shows the good stability of the sensor. The downside is that the GF of the sensor based on Kirigami 2D MoS_2_ is reduced. In order to improve the GF of strain sensor. Zhu et al. [198] used V-doped MoS_2_ to fabricate the piezoresistive sensor. The photolithography and following reactive ion etching process were used to make patterned MoS_2_. Figure 15d shows the optical image of the strain sensor. The properties of the strain sensor were carried out using a four-point bending method (inset in Fig. 15e). The highest GF was measured in 20% V-doped MoS_2_ device, which is up to 140 (Fig. 15f).

2D continuous films are very suitable for fabricate piezoresistive sensors. Feng et al. [200] reported a kind of electronic-skin strain sensor based on CVD-grown 2D In_2_Se_3_ continuous films. Figure 15g shows the macrophotograph and microscope image of the sensor which was fabricated on a PET substrate. The *I*–*V* curves (Fig. 15h) show symmetrically change under the tensile and compressive strain. The calculated GF is 237 and 92 for applied − 0.5–0.5% strain and 0.5–1.5% strain. A stable response to the strain in Fig. 15i shows the good stability and mechanical robustness of the 2D In_2_Se_3_-based strain sensor. Wagner et al. [201] developed a piezoresistive pressure sensor based on CVD-grown 2D PtSe_2_ continuous film. The negative GF of the piezoresistive sensor is up to − 85 obtained experimentally in a bending cantilever beam setup (Fig. 15j). Figure 15k shows the high piezoresistive response to the 2 kg load of 2D PtSe_2_-based strain sensor. The sensitivity of the sensor is higher than the similar sensors based on SWNTs, graphene, GaAs, etc., as shown in Fig. 15l.

A few strain sensors were also reported based on the piezoelectric effect of 2D semiconductors. Qi et al. [170] demonstrated that the highest GF of monolayer MoS_2_ piezoelectric strain sensors was 1160, which is much greater than that of similar 2D semiconductor-based sensor, while Song et al. [202] showed that the GF of 2–3 nm PbI_2_ piezoelectric strain sensors was only 10–25. So far, the publications about the properties of 2D materials piezoelectric strain sensors is still very limited and still needs further study.

A list of strain sensor based on 2D semiconductors and their properties is shown in Table 2. The GF values of some piezoresistive force sensors based on 2D semiconductors are higher than state-of-the-art silicon-based strain sensors and much higher than the graphene-based strain sensors. More importantly, the strain sensors based on 2D semiconductors are flexible, are transparent and can withstand larger strain compared with conventional silicon sensors. 2D crystal quality directly affects device performance, so the development of preparation technology of 2D materials is crucial for its application in the field of strain sensors. High performance, functionalization and integration are the development direction of strain sensors.

**Table 2:** A list of strain sensor based on 2D materials and their properties

Materials	Thickness	Method	Size	Strain (%)	GF	Refs.
MoS_2_	1 L	Mechanical exfoliation	10–20 μm	Tensile	148	[171]
MoS_2_	2 L	Mechanical exfoliation	10–20 μm	Tensile	224	[171]
MoS_2_	3 L	Mechanical exfoliation	10–20 μm	Tensile	43.5	[171]
MoS_2_	3 L	Mechanical exfoliation	10–30 μm	0–20	240	[197]
MoS_2_	2 L	CVD	Large area	− 2–2	56.5/72.5	[196]
MoS_2_	7 nm	CVD	Large area	Tensile and compress	140	[198]
MoS_2_	1 L	CVD	Large area	0–15	3.5	[199]
MoS_2_	1 L	CVD	Large area	0–0.03	104	[198]
BP	20 nm	Mechanical exfoliation	10–30 μm	− 0.15–0.13	~185	[132]
PtSe_2_	4.5–9 nm	CVD	Large area	0–0.04	84.8	[201]
In_2_Se_3_	0.8–3.1 nm	CVD	Large area	−0.5–0.5 0.5–1.5	∼237 ~92	[200]
MoS_2_	1 L	CVD	20 μm	0–0.047	1160	[170]
PbI_2_	2–3 nm	CVD	Large area	0.339	10–25	[202]
SnSSe	1 L	CVD	35 μm	0.9%	69.7	[133]

### Tuning the Performance of Photodetector

#### Tuning the Performance of Photodetector by Piezoelectric Effect

Strain-induced piezoelectric effect had been proved an effective method to regulate the performance of photodetector based on nanowires, nanoribbons and nanofilms. After observing the piezoelectric properties of 2D materials, their applications in 2D optoelectronic devices have also been studied successively. Wu et al. [203] showed the piezophototronic effect which is the first experimental demonstration in monolayer 2D MoS_2_-based flexible optoelectronics. The separation and transport of photogenerated carriers at the interface of MoS_2_–metal can be modulated effectively, which is attributed to the piezoelectric polarization charges created at the two-terminal interface by applying strain. Notably, the maximum photoresponsivity of the device reached up to 2.3 × 10^4^ A W^−1^ by a compressive strain of − 0.38%, with a 26-fold improvement over the reported highest photoresponsivity for single-layer MoS_2_ phototransistors. Recently, the analogous experiment was also performed on 2D multilayer γ-phase InSe photodetector. The responsivity and response speed of this photodetector were enhanced further by as much as 696% and 1010% when the device was subjected to a 0.62% uniaxial tensile strain due to the piezo-phototronic effect [204].

**Fig. 16:**
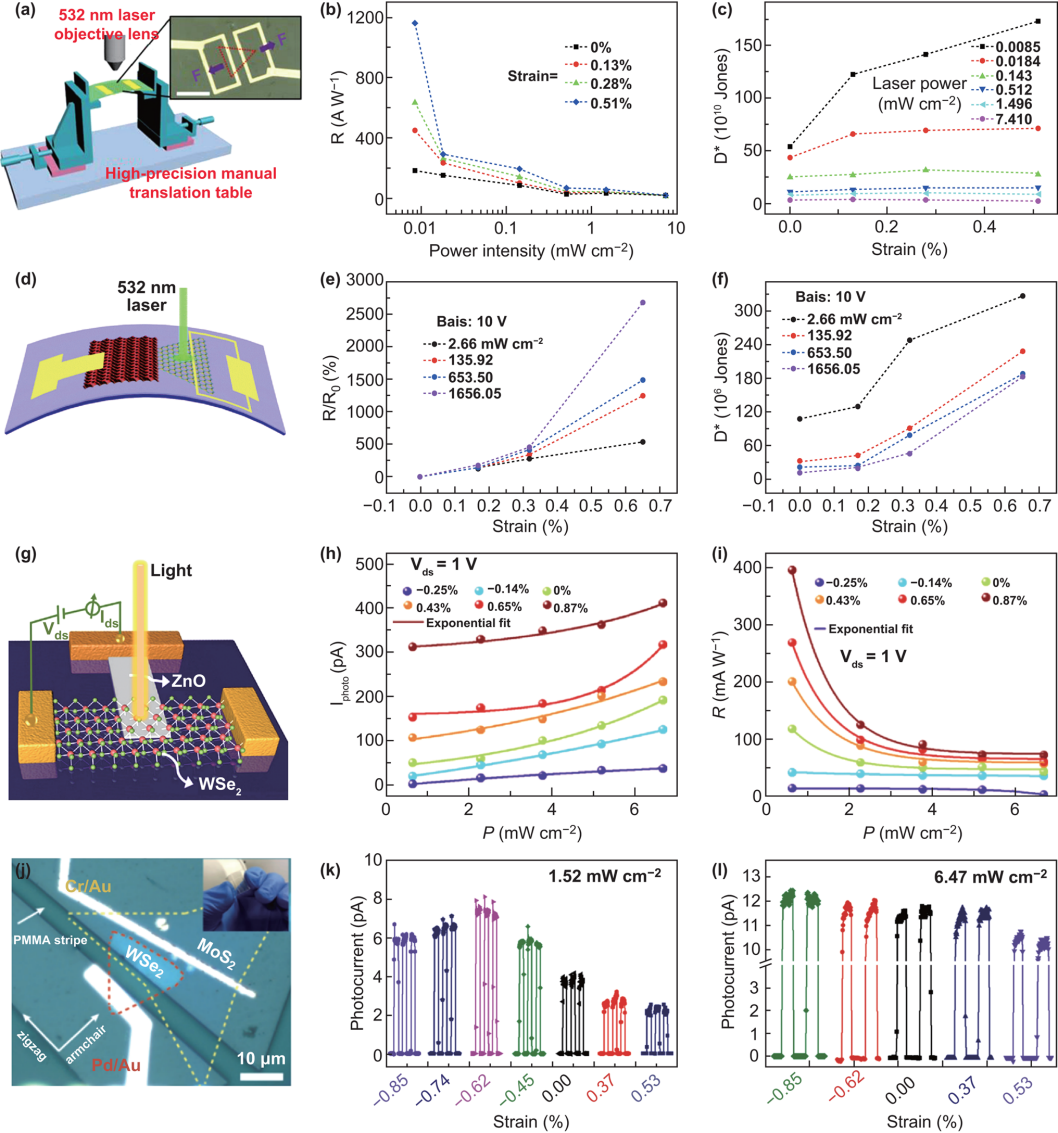
Tuning the performance of photodetector by strain-induced piezoelectric effect. **a** Home-made high-precision table for loading strain. The optical image presents a MoS_2_ device structure. Variations in value of **b** photoresponsivity and **c** detectivity at a bias of − 10 V under different strains and various optical power intensities. Reproduced with permission [205]. Copyright 2018, IOP Publishing. **d** Schematic diagram of the flexible photodetector based on the *p*-CuO/*n*-MoS_2_ heterojunction. The strain dependence of **e** *R*/*R*_0_ and **f** detectivity at different power densities at a bias of 10 V. Reproduced with permission [206]. Copyright 2017, Royal Society of Chemistry. **g** Schematic diagram of WSe_2_–ZnO photodetector on flexible PET substrate. **h** Strain-dependent photocurrent and **i** responsivity of the device for different illumination intensities under 1 V applied external bias. Reproduced with permission [207]. Copyright 2016, Elsevier. **j** Optical image of the fabricated MoS_2_/WSe_2_ *p*–*n* photodiode device. Strain dependence of photocurrent in the device under the illumination of **k** 1.52 mW cm^−2^ and **l** 6.47 mW cm^−2^ light at zero applied bias. Reproduced with permission [208]. Copyright 2018, Wiley–VCH

Zhang et al. [205] designed and fabricated a flexible photodiode based on single-layer MoS_2_ lateral *p*–*n* homojunction by chemical doping (Fig. 16a), and piezo-phototronic effect was used to enhance the photocurrent. The device presented significant improvement in the photoresponsivity and detectivity for an external tensile strain at 0.51%. The photoresponsivity was enhanced by 619%, from 189 to 1162 A W^−1^ (Fig. 16b), and detection sensitivity was also improved by 319%, up to 1.72 × 10^12^ Jones (Fig. 16c). Meanwhile, a flexible photodetector fabricated by a *n*-MoS_2_/*p*-CuO heterojunction is also demonstrated by their group, the method as shown in Fig. 16d [206]. Because the depletion region can be broadened by piezo-potential under strain, the responsivity of the device under illumination is up to 27 times by a 0.65% tensile strain (Fig. 16e) compared to the device without strain. Besides, the maximum detectivity can reach up to 3.27 × 10^8^ Jones in low light illumination (Fig. 16f). Du et al. [207] proposed a distinctive strain-gating method, which utilized piezo-polarization charges induced by the surface of ZnO nanobelt as “gate” to modulate the photogenerated carriers separation and transport at the vdWs interface of ZnO–WSe_2_. With the increase in tensile strain, the photocurrent of the device displayed an increase from 61 to 320 pA (Fig. 16h), and the corresponding photoresponsivity of the device also showed an improvement, from 117 to 394 mA W^−1^ under white light illumination (Fig. 16i). Lin et al. [208] fabricated a flexible vdWs *p*–*n* photodiode by vertically stacking a few-layer *p*-WSe_2_ and single-layer *n*-MoS_2_ (Fig. 16j). The results demonstrated that the photocurrent of the heterojunction increased by 86%, and a maximum photoresponsivity reached up to ≈ 3.4 mA W^−1^ (Fig. 16k) under a low illumination power density of 1.52 mW cm^−2^ and a compressive strain of − 0.62% along the armchair direction of MoS_2_, while the photocurrent of the device increased only by 6.1% under a higher illumination density of 6.47 mW cm^−2^ (Fig. 16l), which originates from the change in light absorption and the strain-induced piezoresistive effect in 2D materials. In short, the piezo-potential can be induced by applying external strain-based piezoelectric effect, which can adjust the band structure at the interface of *p*–*n* homojunction or heterojunction; as well, the depletion region can be also broadened, resulting in a significantly increase in photogenerated carrier separation and transport. Thus, the optical sensing performance is enhanced effectively. In addition, the carrier concentration *N*_D_ and *N*_A_ in 2D materials increases with a high illumination intensity, which leads to a strong screening effect of piezoelectric polarization (*Q*_piezo_) charges and a lower density of effective *Q*_piezo_. Therefore, the modulation of strain on optical sensing performance are significant differences between a high illumination intensity and low optical intensity.

The piezo-phototronic effect in piezoelectric nanomaterials (thin film and nanowire) was also used to regulate the interface characteristics between 2D material and them, resulting in improving the photoelectric properties of the heterostructures, such as ZnO-MoS_2_ [209], GaN-MoS_2_ [210], and CdS-WSe_2_ [211] heterostructures.

Table 3 displays a list of piezoelectricity enhancing the performance of the photodetectors constructed by 2D semiconductors and their heterostructures. The positive and negative value of strain is uniaxial tensile and compressive strain, respectively. The increased column lists the percent increased of piezoelectricity enhanced photoresponsivity compared with unstrained condition. The photodetector can be divided into two categories, which is Schottky diode and heterojunction device. Piezoelectricity can modulate the Schottky barrier of MS contacts or the depletion zone width of heterojunctions. The improved photo-response performance is due to the separation and transport of photo-generated carriers at the interface of MS or heterojunction were enhanced by strain-induced piezopolarization. Different interfaces require different piezopolarization directions to enhance their performance and the direction of polarization depends on the applied strain type (tensile or compressive), while the best photo-response performance of 2D materials by piezo-phototronic effect is achieved under about ± 0.6% strain. This is easy to implement, but it also limits the possibility of further improving the photoelectric performance by applied large strain. In principle, the piezoresistive effect can also modulate the Schottky barrier of MS contacts and heterosturctures. However, there are less experimental work reported that the performance of photodetectors was tuned by piezoresistive effects in 2D semiconductors.

**Table 3:** A list of piezoelectricity enhancing the performance of the photodetectors constructed by 2D semiconductors and their heterostructures

Device	Strain (%)	Illumination density	Photoresponsivity	Increased (%)	Refs.
MoS_2_	− 0.38	3.4 μW cm^−2^	2.3 × 10^4^ A W^−1^	178	[203]
γ-InSe	0.62	0.368 mW cm^−2^	198.2 mA W^−1^	696	[204]
MoS_2_/MoS_2_	0.51	8.5 μW cm^−2^	1162 A W^−1^	619	[205]
MoS_2_/WSe_2_	−0.62	1.52 mW cm^−2^	3.4 mA W^−1^	86	[208]
ZnO/WSe_2_	0.78	0.667 mW cm^−2^	394 mA W^−1^	236	[207]
WSe_2_/CdS	−0.73	16.9 μW cm^−2^	33.4 A W^−1^	110	[211]
CuO/MoS_2_	0.65	1656 mW cm^−2^	–	2700	[206]

#### Tuning the Performance of Photodetector by Piezoresistive Effect

**Fig. 17:**
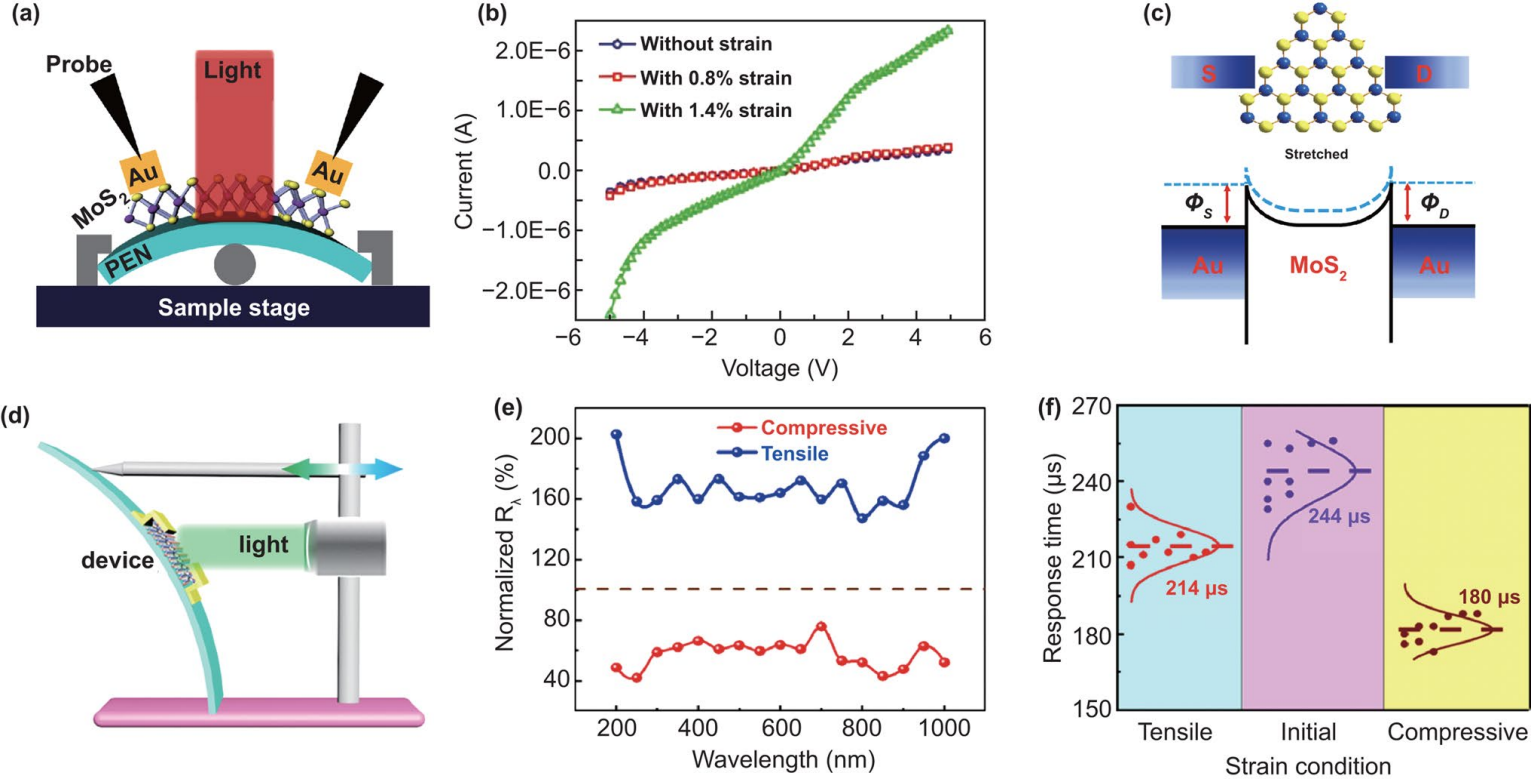
Tuning the performance of photodetector by strain induced piezoresistive effect. **a** Schematic illustration for photoelectric performance measurement of monolayer MoS_2_ photodetector under strain. **b** *I*–*V* characteristics under light illumination and different strains. **c** Band diagram of the monolayer MoS_2_ photodetector under 0.8% strain. Reproduced with permission [212]. Copyright 2019, Wiley–VCH. **d** Schematic diagram of the In_2_Se_3_ photodetector with applied strain. **e** Photoresponsivity of In_2_Se_3_ under different strain. **f** Response time under different strain. Reproduced with permission [214]. Copyright 2019, American Chemical Society

Recently, for the first time, we have proved that not only the piezoelectric effect strain but also the piezoresistive effect caused by strain can improve the performance of the monolayer MoS2 photodetector, the measurement setup as shown in Fig. 17a [212]. The results show that the photo-response speed, light–dark current ratio and self-powered current are improved by small strain (0.8%) due to the piezoelectric effect. The *I***–***V* curves in Fig. 17b shows that the photocurrent of the photodetector was obviously enhanced under 1.4% strain. And the photoresponsivity of the device is significantly improved by relatively large strain (1.4%) thanks to the piezoresistive effect. The photocurrent increases from 0.37 to 2.35 μA and photoresponsivity increases from and 114.3 to 590.0 A W^**−**1^. Analogously, the strain-induced non-linearity increase in photocurrent and photoresponsivity of monolayer MoS_2_ devices was also reported by Gant et al. [213]. Lately, Li et al. [214] reported that the photoresponsivity and photo-response speed of a 3.2-nm In_2_Se_3_ can be synchronously improved by piezoresistive effect, the method of which is shown in Fig. 17d. Figure 17e, f shows the device under 0.65% tensile strain, the photoresponsivity increase from 0.22 to 0.37 A W^**−**1^ and the photo-response time decrease from 244 to 214 μs. Dai et al. [204] demonstrated the photocurrent of β-InSe multilayers-based photodetector achieved a 211% enhancement ratio under a uniaxial tensile strain of 0.62% due to piezoresistive effect.

The piezoresistive effect in 2D semiconductors can reduce the barrier heights of two Schottky junctions simultaneously (Fig. 17c) due to the narrowed band gap and induced carrier density improvement. The narrowed band gap broadens the absorbed light’s wavelengths and the reduced barrier heights improved the photocurrent, then the photoresponsivity is increased. Gant et al. [213] believe that the increased long-lived charge traps in sample can also explain the large increase in responsivity. In addition, the direct to indirect band gap transition in any 2D semiconductors will also affect their photo-response performance.

Table 4 displays a list of piezoresistivity-enhanced photo-response performance of 2D semiconductors. It is clear that the strain-induced piezoresistive effect can effectively improve the photocurrent and photoresponsivity of the device. One of these studies shows that the photo-response speed can also be improved. Because the piezoresistive effect is universal in semiconductors, the above significant studies are beneficial to the application of 2D semiconductors in optoelectronics.

**Table 4:** A list of piezoresistivity-enhanced performance of the photodetectors constructed by 2D semiconductors

Device	Strain (%)	Illumination density (mW cm^−2^)	Photoresponsivity	Increased (%)	Refs.
MoS_2_	1.4	50	590.0 A W^−1^	354	[212]
MoS_2_	0.48	12	61 mA W^−1^	>10,000	[213]
In_2_Se_3_	0.65	0.77	0.37 A W^−1^	68	[214]
β-InSe	0.62	0.368	52.5 mA W^−1^	111	[204]

### Nanogenerators

Recently, study on energy harvesting used nanomaterials has attracted more and more attention on solving the energy and environmental problems. Nanogenerator (NG) fabricated by piezoelectric nanomaterials is an effective energy-harvesting technology through converting mechanical energy into electrical energy which have normally no restrictions, are user-friendly and are ubiquitous [215]. In 2014, the work of Wu et al. [39] shows that 2D materials have potential applications for building nanogenerators. The mechanically exfoliated monolayer MoS_2_ can generate 15 mV and 20 pA output when applying 0.53% strain. The output of CVD-grown monolayer is lower, while the voltage and current outputs can be enhanced by serial connection and parallel connection, respectively. Soon afterward, theoretical study of odd-layer MoS_2_ NGs shows that outputs and energy conversion efficiency are decreased with the increasing thickness of MoS_2_. Compare with the NGs-based nanowires and nanofilms, the outputs of 2D MoS_2_-based NGs are relative small [216].

**Fig. 18:**
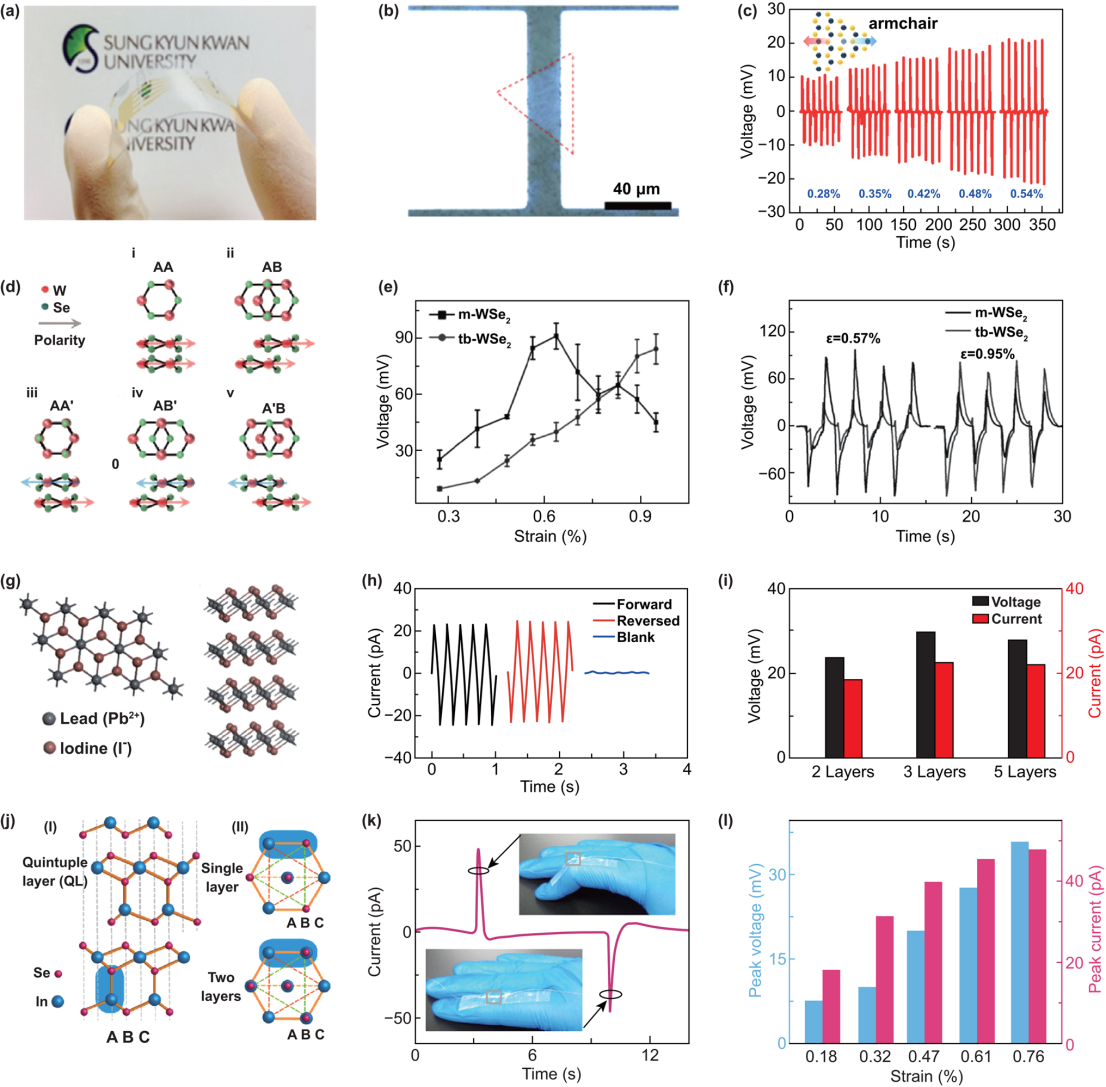
Piezoelectric generators based on 2D semiconductors **a** Photographic image of the CVD-grown monolayer MoS_2_-based flexible piezoelectric NG. **b** Optical images showing energy harvesting active regions with the armchair atomic orientations of the piezoelectric NG. **c** Output voltage obtained from the monolayer MoS_2_ NG under different applied strain. Reproduced with permission [176]. Copyright 2016, Elsevier. **d** Stacking structure for bilayer-WSe_2_. **e** Piezoelectric peak output voltages of monolayer WSe_2_ and bilayer WSe_2_ as a function of strain. **f** Piezoelectric output voltage of m-WSe_2_ (black line) and tb-WSe_2_ with a low strain. Reproduced with permission [217]. Copyright 2017, Wiley–VCH. **g** Crystal structure of monolayer and multilayer PbI_2_. **h** Piezoelectric output current of 2D PbI_2_ device. **i** Piezoelectric peak output voltages of devices fabricated with 2, 3, and 5 layers PbI_2_ nanosheets. Reproduced with permission [218]. Copyright 2018, American Chemical Society. **j** Atom structure of hexagonal α-In_2_Se_3_. **k** An α-In_2_Se_3_ piezoelectric nanogenerator is integrated on an index finger to scavenge the mechanical energy induced by finger motions. **l** Strain dependence of the piezoelectric voltage output. Reproduced with permission [218]. Copyright 2018, American Chemical Society

In 2016, the flexible piezoelectric nanogenerators based on CVD-grown monolayer MoS_2_ were fabricated by Kim et al. [176], the photographic image and optical images as shown in Fig. 18a, b. The results show that the piezoelectric output power can be generated from the NGs with both armchair and zigzag direction of monolayer MoS_2_, while the output power of armchair direction NG is much higher than that of the zigzag direction NG under the same test conditions. Under 0.54% strain in armchair direction, the measured output piezoelectric voltage and current are over 20 mV (Fig. 18c) and over 30 pA.

Lee et al. [217] found the manually stacked twisted bilayer WSe_2_ (stacked by two-layered CVD-grown monolayer WSe_2_) has reliable piezoelectric properties. DFT simulation results show that the AA- and AB-stacked bilayer WSe_2_ has relatively high piezoelectric coefficients due to its non-centrosymmetry crystal structure (Fig. 18d). The experiment results show that the output voltage of bilayer WSe_2_ NGs is continuously increased to 85 mV with strain increase to 0.95%, while the output voltage of monolayer WSe_2_ NGs is up to a maximum value of 90 mV when applying 0.64% (Fig. 18e, f).

Song et al. [202] reported a piezoelectric nanogenerator based on CVD-grown 2D PbI_2_ nanosheets. Figure 18g shows the crystal structure of PbI_2_. Figure 18h, i shows the piezoelectric current output of a 2-nm-thickness PbI_2_ NG is about 20 pA. The output voltage of different thicknesses PbI_2_ NG suggests that the piezoelectricity is not affected by the number of layers. The max output voltage is 29.4 mV.

The piezoelectric coefficient of *d*_33_ (out-of-plane) and *d*_11_ (in-plane) coexists in 2D α-In_2_Se_3_ without thickness effect due to its non-centrosymmetric crystal structure (Fig. 18j). Xue et al. [218] developed flexible piezoelectric NG based on multilayer α-In_2_Se_3_, with a max output current of 47.3 pA and voltage of 35.7 mV, respectively, under applied 0.76% strain (Fig. 18l). They integrated the NG onto human skin, and the results (Fig. 18k) indicate its application in energy harvesting and electronic skin.

Lately, a flexible NG based on multilayer BP was reported for the first time by Du et al. [219]. The in-plane piezoelectricity is experimentally observed in multilayer BP along the armchair direction. The *I*–*V* curves of the device under different strains suggest a piezotronic effect. The intrinsic current output is about 4 pA under − 0.72% compressive strain. Obviously, the output of BP NG is not very well in the similar device, while the piezoelectric effect in BP can help to improve its infrared detection performance.

It is well known that there are intrinsic S vacancies unavoidably existing in 2D MoS_2_, especially for the samples prepared by CVD methods, resulting in the lower output of NG based on CVD-grown MoS_2_. As discussed above, Han et al. [220] reported improving the performance of NGs based on CVD-grown monolayer MoS_2_ by passivating S vacancy. They found that the electron concentration is a key role in effecting the output of NGs. The S vacancies can be passivated effectively by employing the process of sulfur vapor treatment. S-treatment reduces the electron concentration of the monolayer MoS_2_ and enhances the piezoelectric effect. The results show that the output of the S-treated monolayer MoS_2_ NGs is increased, the max output current reaches up to 100 pA (increased by over three times) and the max output voltage is 22 mV (increased by two times). An increasing output piezoelectric current was investigated with the increase in strain rate. Interestingly, Dai et al. [221] found that grain boundaries in monolayer MoS_2_ can significantly enhance its piezoelectric property. The output power of piezoelectric nanogenerator made of the CVD-grown butterfly-shaped monolayer MoS_2_ was 50% higher than the device made of triangular sample. The improved piezoelectricity is attributed to the additional piezoelectric effect induced by the deformable grain boundaries, which can promote polarization and generate spontaneous polarization with different piezoelectric coefficients along various directions. These results indicate that the piezoelectric performance of 2D semiconductor can be improved by controlling the defects, not only repairing defects but also utilizing defects, which is significance for further applications.

In this section, we reviewed the applications of strain-engineered 2D semiconductors in strain sensors, photodetectors and nanogenerators. Most of strain sensors are based on piezoresistive effects and few based on piezoelectric effect. The GF values of some piezoresistive strain sensors based on 2D semiconductors are higher than state-of-the-art silicon-based strain sensors and can withstand larger strain than traditional commercial strain sensors. 2D crystal quality directly affects device performance, so the development of preparation technology of 2D materials is crucial for its application in the field of strain sensors. So far, the performance of photodetectors based on 2D semiconductor can be improved by piezoelectricity reduction in the Schottky barrier of MS contacts or widening the depletion zone of PN junctions. Some latest results also show that the photocurrent and photoresponsivity of a monolayer MoS_2_ photodetector are improved significantly by strain-induced piezoresistive effect. The two effects synergistically modulate the photo-response properties of the 2D materials which is worthy of attention. The output of piezoelectric nanogenerator based on 2D materials is relatively small, and its application prospects are worth pondering.

## Summary and Perspective

The mechanical strain in material can be used to develop advanced strain sensors and energy harvesting devices, and the strain can also alter other physical and chemical characteristics of the material itself, such as electricity, optics, surface state, adsorption, catalysis and magnetism, which have great potential in materials science, condensed matter physics, electronic science and technology, chemistry, biology, and even quantum mechanics, etc. The strain engineering has been successfully used for improving the performance of the transistors based on traditional semiconductors. However, it is still in infancy stage for 2D materials. Compared with bulk and 1D materials, 2D materials have a simpler structure, smaller size, better flexibility, higher transparency, stronger performance adjustability and easier to pattern and manufacture-integrated devices for large-area continuous 2D materials which are promising for high-performance electronics and optoelectronics. On basis of large-simulation studies of strain in 2D semiconductors, the piezoresistive and piezoelectric effect was experimentally investigated began 2013 and 2014, respectively. After the worldwide constant efforts in recent years, the research of strain in 2D semiconductors has made a rapid progress.

Herein, we comprehensively review the recent progress of strain-induced piezoelectric and piezoresistive effect-engineered 2D semiconductors in three aspects: The basic theories and simulation studies of piezoelectric and piezoresistive effect in different 2D semiconductors, engineering methods, such as fabricating flexible device, employing stretchable and patterned substrate, creating wrinkles and using AFM apparatus, for introduced strain in 2D semiconductors, and the applications of strain-engineered 2D semiconductors in strain sensors, nanogenerators and tuning the performance of photodetectors. The fundamentals and applications of strain-engineered 2D semiconductors offer exciting opportunities as well as facing great challenges; the efforts may be made in the following aspects:

1. The piezoelectricity in 2D TMDs is limited to in-plane specific direction and odd number layers, which makes it to be limited in applications. With the expansion of the 2D materials, such as wurtzite structure, Janus 2D materials and monolayer SnSe which have been predicted to process highest piezoelectric coefficient, their piezoelectric properties urgently need to be observed experimentally.
2. According to the results of calculations, the piezoresistive effect is conducive to the photo-response performance of the 2D semiconductors due to the variation of band gap, absorption spectrum and PCE performance. Four experimental studies reported in 2019 showed some inspiring results, as shown in Table 4, while the studies in this aspect are still lacking, especially for 2D van der Waals heterostructures which have not been reported. Since the piezoresistive effect is universal in semiconductors, such studies are beneficial to the application of 2D semiconductors in optoelectronics. Lots of works suggested that the photo-response performance of 2D semiconductors or 2D heterostructures can be improved by piezoelectric effect, but rarely consider the piezoresistive effect. The two effects synergistically modulate the photo-response properties of the 2D materials are worthy of attention, and further study mechanism and argumentation are necessary.
3. Although a variety of methods for applying strain on 2D materials have been developed, most of them are limited to experimental observation and verification, while not suitable for the applications of electronic components. The new techniques, which can permanently and easily integrate applied strain on the devices based on 2D semiconductors, need to be developed to permanently modulate their properties for developing high performance transistors. In addition, the service performance of the devices based on strained 2D materials is also very important.
4. High-quality and large-scale 2D crystal preparation technology with low costs is still the essential to promoting its industrial application. The core technology of controlled synthesized 2D semiconductors with stable structure and properties is inadequate. So far, the large-sized some 2D materials can be synthesized by CVD method, but inevitably with a large number of defects, such as vacancies and grain boundaries. The recent studies show that the output of nanogenerator can be enhanced by repaired vacancies or utilized grain boundaries. However, studies on strain-engineered defective 2D semiconductors in strain sensors and photodetectors are inadequate. Predictable, the performance of the strain sensor will be modulated by controlling the defects. In addition, the elegant integration of force sensors or nanogenerators can drive their applications. Multifunction of the strain sensors based on 2D materials is also a promising research direction.
5. Strain-engineered 2D materials also show application prospects in the area of gas sensors, catalysis and spintronics. Particularly, the cutting-edge topics of quantum orderings, such as superconductivity and ferroelectricity in 2D materials, can also be engineered by strain. Strain engineering has fascinating potential in the condensed matter physics, such as control quantum Hall effect, topological states and chiral fermions, which may bring about new physics and novel devices.
